# Sulfite dynamics affect drought tolerance and water status in Arabidopsis and tomato

**DOI:** 10.1093/jxb/erag344

**Published:** 2026-07-10

**Authors:** Kusum Khatri, Jaykumar Patel, Aizat Bekturova, Poonam Tiwari, Tesfaye Asmare Sisay, Babita Choudhary, Dominic Standing, Beery Yaakov, Simon Barak, Veronika Turečková, Miroslav Strnad, Moshe Sagi

**Affiliations:** Jacob Blaustein Center for Scientific Cooperation, The Jacob Blaustein Institutes for Desert Research, Ben-Gurion University of the Negev, Sde Boker Campus, Midreshet Ben-Gurion 8499000, Israel; Department of Life Sciences, Aberystwyth University, Penglais, Aberystwyth, SY23 3DA, UK; Jacob Blaustein Center for Scientific Cooperation, The Jacob Blaustein Institutes for Desert Research, Ben-Gurion University of the Negev, Sde Boker Campus, Midreshet Ben-Gurion 8499000, Israel; Institute of Biological, Environmental & Rural Sciences (IBERS), Aberystwyth University, Gogerddan, Aberystwyth, SY23 3EE, UK; Jacob Blaustein Center for Scientific Cooperation, The Jacob Blaustein Institutes for Desert Research, Ben-Gurion University of the Negev, Sde Boker Campus, Midreshet Ben-Gurion 8499000, Israel; Jacob Blaustein Center for Scientific Cooperation, The Jacob Blaustein Institutes for Desert Research, Ben-Gurion University of the Negev, Sde Boker Campus, Midreshet Ben-Gurion 8499000, Israel; The Albert Katz International School for Desert Studies, The Jacob Blaustein Institutes for Desert Research, Ben-Gurion University of the Negev, Sde Boker Campus, Midreshet Ben-Gurion 8499000, Israel; Jacob Blaustein Center for Scientific Cooperation, The Jacob Blaustein Institutes for Desert Research, Ben-Gurion University of the Negev, Sde Boker Campus, Midreshet Ben-Gurion 8499000, Israel; The Albert Katz Department of Dryland Biotechnologies, French Associates Institute for Agriculture and Biotechnology of Dryland, The Jacob Blaustein Institutes for Desert Research, Ben-Gurion University of the Negev, Sde Boker Campus, Midreshet Ben-Gurion 8499000, Israel; The Albert Katz Department of Dryland Biotechnologies, French Associates Institute for Agriculture and Biotechnology of Dryland, The Jacob Blaustein Institutes for Desert Research, Ben-Gurion University of the Negev, Sde Boker Campus, Midreshet Ben-Gurion 8499000, Israel; The Albert Katz Department of Dryland Biotechnologies, French Associates Institute for Agriculture and Biotechnology of Dryland, The Jacob Blaustein Institutes for Desert Research, Ben-Gurion University of the Negev, Sde Boker Campus, Midreshet Ben-Gurion 8499000, Israel; Laboratory of Growth Regulators, The Czech Academy of Sciences, Institute of Experimental Botany, Palacky University, Olomouc CZ-78371, Czech Republic; Laboratory of Growth Regulators, The Czech Academy of Sciences, Institute of Experimental Botany, Palacky University, Olomouc CZ-78371, Czech Republic; The Albert Katz Department of Dryland Biotechnologies, French Associates Institute for Agriculture and Biotechnology of Dryland, The Jacob Blaustein Institutes for Desert Research, Ben-Gurion University of the Negev, Sde Boker Campus, Midreshet Ben-Gurion 8499000, Israel; Katif Research Center, Sedot Negev 8771002, Israel; Ministry of Science and Technology, Netivot 8771002, Israel; Universidade Federal do Ceará, Brazil

**Keywords:** Abscisic acid, cysteine, drought, sulfate, sulfite, water loss

## Abstract

Previously, we showed that altered sulfite homeostasis led to higher water loss in *sulfite oxidase* (*SO*) RNA interference (Ri) plants than in Arabidopsis wild type (WT) after sulfite infiltration into rosette leaves. In contrast, *SO* overexpression (OE) and *adenosine-5′-phosphosulfate reductase 2* (*apr2*) knockout (KO) resulted in lower water loss than in WT. Accordingly, sulfite homeostasis and drought tolerance under prolonged drought in Arabidopsis and tomato (Rheinlands Ruhm) were investigated. *SO* Ri mutants displayed the most severe wilting and water loss, accompanied by sulfite accumulation due to reduced *SO* expression. In contrast, *SO* OE, *apr2* KO, *apr2* KO/*SO* OE, and gene-edited lines, including *sulfite reductase* (*SiR*) OE*^C2^*+*SO* OE*^C2^* and *SiR* OE*^C4^*/*SO* OE, maintained higher relative water content by enhancing sulfite oxidation and reduction. Elevated sulfite in *SO* Ri mutants acted as an antagonist to abscisic acid (ABA), as despite accumulating higher ABA than all other genotypes, *SO* Ri plants displayed the lowest relative water content, consistent with sulfite suppressing ABA signal transduction through impaired by Pyrobactin resistance 1-like 5 (PYL5) and enhanced ABA negative feedback by Highly ABA-induced PP2C gene 2 (HAI2). *SO* and *SiR* overexpression also increased hydrogen sulfide and cysteine levels, contributing to drought resilience. Tomato lines also reflected these patterns in *SO* OE, *SiR* OE, and *SiR* OE/*SO* OE, showing reduced sulfite and improved relative water content compared with WT. Overall, coordinated regulation of SO, SiR, and APR2 maintains sulfite homeostasis, mitigating drought-induced damage and enhancing water retention across species by directly limiting sulfite accumulation and relieving sulfite-driven antagonism of ABA signal transduction.

## Teaser

Regulating sulfite via a modified sulfate reduction pathway alters water content in Arabidopsis and tomato, linking sulfur metabolism to plant hydration and suggesting potential strategies to enhance drought tolerance.

## Introduction

Sulfur (S) is one of the most essential macronutrients (constituting 0.1% of dry matter) in living systems for catalytic and electrochemical functions, and it can be present as sulfate (SO_4_^2−^), sulfite (SO_3_^2−^), elemental sulfur (S^0^), or sulfide (H_2_S). Additionally, sulfur is also present in functional groups of metabolites with diverse and specialized biological roles, such as cysteine, methionine, glutathione, phytochelatins, biotins, molybdenum cofactor, thiamine, coenzymes, *S*-adenosyl-methionine, metallothioneins, and glucosinolates (Leustek, 2002). The uptake of available sulfate from the soil system is essential for plant growth and development (Takahashi *et al*., 2011). The utilization of sulfate for synthesizing S-containing metabolites and uptake from the soil is optimized by regulating the membrane transport system and metabolic enzymes involved in S acquisition, storage, and utilization (Takahashi *et al*., 2011; Kopriva *et al*., 2012; Takahashi, 2019).

Sulfite assimilation in plants starts with converting sulfate to adenosine-5′-phosphosulfate (APS) by ATP sulfurylase (EC 2.7.7.4) in the chloroplast and cytosol. In the next step, APS is reduced to sulfite by the plastid-localized APS reductase enzymes [APR; EC 1.8.4.9] (Kopriva and Koprivova, 2004; Takahashi *et al*., 2011). Above a certain level, the generated sulfite ions can be toxic and disrupt metabolic processes, such as photosynthesis and respiration (interfering with thiol/disulfide functional groups), inactivating proteins (thioredoxins), and causing lipid peroxidation (cell membrane damage) due to their nucleophilic nature (Peiser and Yang, 1985; Würfel *et al*., 1990; Bekturova *et al*., 2021). Sulfite can be accumulated by atmospheric SO_2_ uptake by foliage and can dissociate in the mesophyll apoplast to bisulfite (HSO^3−^) and sulfite (SO_3_^2−^; Yi and Meng, 2003). Additionally, sulfite can accumulate due to the degradation of the S-amino acids cysteine and methionine (Hänsch and Mendel, 2005; Brychkova *et al*., 2013, 2015; Kurmanbayeva *et al*., 2022).

Sulfite is oxidized to sulfate by sulfite oxidase (SO; EC 1.8.3.1), the smallest molybdenum-containing enzyme localized in the peroxisomes. During this process, hydrogen peroxide (H_2_O_2_) is generated by the transfer of two electrons, first to the molybdenum cofactor redox center and then to molecular oxygen (Eilers *et al*., 2001). Among macronutrients, sulfate is the only one that has been shown to increase in the plant xylem sap during drought stress. This precedes increases in the biosynthesis of abscisic acid (ABA), essential for inducing stomatal closure, and sulfate (i) induces the function of 9-*cis*-epoxycarotenoid dioxygenase 3 (NCED3), the committed step of ABA biosynthesis, and (ii) is incorporated into cysteine, which is exploited in the sulfuration of the active center of Arabidopsis aldehyde oxidase 3, the enzyme that catalyses the oxidation of abscisic aldehyde to ABA (Sagi *et al*., 1999, 2002; Ernst *et al*., 2010; Malcheska *et al*., 2017; Batool *et al*., 2018).

Sulfite is reduced to sulfide (H_2_S) by chloroplast-localized sulfite reductase (SiR; EC 1.8.7.1) activity, employing NADPH-originated electron transport generated by the FAD-containing and chloroplast-localized ferredoxins (Yarmolinsky *et al*., 2013, and references therein). Further, at the end of the sulfate reduction pathway, sulfide, along with *O*-acetyl serine, is converted to cysteine by *O*-acetyl serine (thiol)lyase (OASTL; Takahashi *et al*., 2011). The sulfide, which plays a role in drought tolerance by controlling stomatal aperture and senescence-triggered autophagy (Álvarez *et al*., 2012; Jin *et al*., 2013; Scuffi *et al*., 2014; Malcheska *et al*., 2017), originates not only from SiR activity but also from L-cysteine degradation by L-cysteine desulfhydrase 1 (Álvarez *et al*., 2010; Romero *et al*., 2014), as well as from L-cysteine degradation by the three OASTLs in Arabidopsis (Kurmanbayeva *et al*., 2022).

Adenosine-5′-phosphosulfate reductase (APR) and SO coordination, which balances the sulfate-sulfite cycle (Randewig *et al*., 2012; Bekturova *et al*., 2021), together with SiR and SO detoxification of excess sulfite, has been demonstrated in Arabidopsis and, in part, in tomato (Brychkova *et al*., 2007; Lang *et al*., 2007; Randewig *et al*., 2012; Yarmolinsky *et al*., 2013). The effects of short-term exposure to sulfite on the sulfite network, that is, APR and SO, the major sulfite-producing and consuming enzymes, and their implications for stomatal aperture and water loss have recently been shown (Bekturova *et al*., 2021). Specifically, sulfite infiltration into the rosette leaves of 23-day-old Arabidopsis plants resulted in significantly higher (*P*<0.05) water loss in *SO* RNA interference (Ri) plants than in wild type (WT), *adenosine-5′-phosphosulfate reductase 2* (*apr2*) knockout (KO), *glutathione reductase 2* (*gr2*) KO, or *SO* overexpression (OE) Arabidopsis rosette leaves. Stomatal apertures of epidermal peels taken from WT and *SO* Ri untreated leaves and incubated with stomatal-opening buffer containing sulfate and/or sulfite revealed that the interplay between sulfate and sulfite levels plays a role in the rate of stomatal opening and water loss; sulfite enhancement increases stomatal opening and enhances water loss, while elevated sulfate decreases stomatal aperture and water loss. Additionally, root detachment enhanced sulfite accumulation in the *SO* Ri rosette leaves, resulting in higher water loss than in the WT, *SO* OE, and *gr2* KO rosette leaves. Significantly, preincubation of detached WT or *SO* Ri rosette leaves in buffer containing 8 mM sulfite resulted in higher water loss than the control detached leaves preincubated in buffer with sulfite absent. In contrast, preincubation of the detached rosette leaves in buffer containing 4 mM sulfate significantly decreased (*P*<0.05) water loss in detached leaves 2 h after removal from the buffer, compared with incubation with sulfite. Moreover, a very short irrigation cessation for 48 h in Arabidopsis plants, grown in a limited volume of field-capacity-watered soil, induced a higher sulfite level in leaves, followed by higher water loss in the *SO* Ri leaves than in the WT and *SO* OE leaves (Bekturova *et al*., 2021).

The response of plants to drought stress depends on genotype, drought severity, and drought duration (Hu and Xiong, 2014; Dinneny, 2019; Gul *et al*., 2023). The current study investigated the intricate role of sulfur assimilation pathway components in response to drought stress. This study advances the work of Bekturova *et al*. (2021) to prolonged, physiologically relevant drought stress in both Arabidopsis and tomato, employing a substantially expanded genetic panel including *SO* OE, *SO* Ri, *apr2* KO, *apr2* KO/*SO* OE double mutants, *SiR*-overexpressing lines, CRISPR–Act3.0-generated transcriptional activation lines, and tomato overexpression lines, together with comprehensive ABA catabolite profiling and *in planta* validation of the sulfite–ABA antagonism model under sustained drought. Arabidopsis WT, *SO* Ri, and tomato [*Solanum lycopersicum* (*Sl*)] Rheinlands Ruhm (RR) WT exhibited reduced relative water content (RWC), increased sulfite levels, and lower sulfate and sulfide levels in response to drought stress. Conversely, Arabidopsis *SO* OE, *apr2* KO, *apr2* KO/*SO* OE, and tomato *SO* OE, *SiR* OE, and *SiR* OE/*SO* OE displayed higher sulfate levels, leading to improved RWC. These results highlight the essential roles of SO, APR, and SiR in maintaining sulfite homeostasis and RWC under drought stress.

## Materials and methods

### Plant materials

*Arabidopsis thaliana* ecotype Col-0 (WT) and mutants, including *SO* OE, *SO* Ri (Brychkova *et al*., 2007)], and *apr2* KO (GABI_108G02; Loudet *et al*., 2007), were used in this study. The pART27:LeSiR construct was used to transform Arabidopsis *SO* OE plants in order to generate *SlSiR*-overexpressing lines (Brychkova *et al*., 2012a). The construct was introduced into *Agrobacterium tumefaciens* strain GV3101 by electroporation and transformed into Arabidopsis *SO* OE plants using the floral dip method (Clough and Bent, 1998). Transformed lines were selected by resistance to kanamycin for *SlSiR* OE/*SO* OE plants and confirmed by western blotting for homozygous lines employing specific antibodies for SO and SiR (Brychkova *et al*., 2007; Yarmolinsky *et al*., 2013). For generating the *apr2* KO/*SO* OE double mutant, the *SO* OE construct was inserted into *apr2* KO plants, as was done when generating *SO* OE on a WT background (Brychkova *et al*., 2007). Further information on Arabidopsis and tomato mutants is provided in Supplementary Table S1.

For the double overexpression of the *SiR* and *SO* genes using CRISPR–Act 3.0 system tools, the promoter region of the respective gene was targeted and cloned into gRNA (Supplementary Table S2) entry as per Supplementary Protocol S1. Construct 1, harboring two *SiR* and two *SO* sgRNA, was transformed into a Arabidopsis WT (Col-0) background, whereas construct 2, harboring four *SiR* sgRNA, was transformed into *SO* OE Arabidopsis plants. CRISPR construct 1 (named *SiR* OE*^C2^*+*SO* OE*^C2^*) and construct 2 (named *SiR* OE*^C4^*/*SO* OE) (where C indicates CRISPR and 2 or 4 indicates the number of guide RNA) were validated using the qPCR and SiR and SO-specific antibodies that we had previously used (Brychkova *et al*., 2007, 2012b).

The tomato plants (*S. lycopersicum*) Rheinlands Ruhm (RR) wild type, *SO* OE, and *SiR* OE (Brychkova *et al*., 2007, 2012b) were used for the present study. These mutants were used to generate tomato plants with the double mutant *SiR* OE*/SO* OE by sterilizing the flowers of *SiR* OE with tweezers before the stamens opened. The *SO* OE pollen was used to hand-pollinate the *SiR* OE stamens. Homozygosity detection of the mutants *SiR* OE*/SO* OE, *SO* OE, and *SiR* OE was performed using SO and SiR antibodies.

### Drought stress treatment and assessment of relative water content in Arabidopsis and tomato plants

For Arabidopsis, different genotypes (WT, *SO* OE, *SO* Ri, *apr2* KO, *apr2* KO/*SO* OE, *SlSiR* OE/*SO* OE, and CRISPR constructs (*SiR* OE*^C2^*+*SO* OE*^C2^* and *SiR* OE*^C4^*/*SO* OE) were grown on half-strength Murashige and Skoog (MS) agar plates for 7 d in a growth room under 14 h of light/10 h of darkness, 22 °C temperature, 75–85% relative humidity, and 150 µmol m^−2^ s^−1^ light intensity. For the drought stress, 7 g of a sterile oven-dried low-nutrient soil mixture was placed in each pot (height 5.5 cm, diameter from top 3.5 cm, and diameter from bottom 2 cm). Seedlings were transferred to the plastic pots irrigated to field capacity 7 d after germination. All genotypes were randomly arranged to minimize edge effects. Plants were irrigated twice a week and grown for 16 d in soil under 14 h of light/10 h of darkness, 22 °C, 75–85% relative humidity, and 150 µmol m^−2^ s^−1^ light intensity. The 23-day-old plants were divided into two groups. The control group continued to be watered, whereas the drought group received no water for 5 d. On the sixth day of drought stress, plant material was harvested in liquid nitrogen and transferred to a −80 °C freezer. Fresh rosette leaves were used to measure RWC. Phenotypes were photographed with a Nikon D750 DSLR with an AF-S Micro Nikkor 105 mm 1:2.8 G lens on manual setting with a fixed ISO 1600, Aperture F/16, and a shutter speed of 1/60 s (Nikon Corp., Japan).

Ten-day-old tomato plants (RR, *SO* OE, *SiR* OE, and *SiR* OE/*SO* OE) were grown in the growth room in pots filled with 25 g of low-nutrient soil (height 6.5 cm, diameter from top 6 cm, and diameter from bottom 4.3 cm; average) and irrigated every other day, over a period of 20 d, to the soil’s field capacity level; then, the 30-day-old plants were divided into the control and the drought-exposed group. The control plants continued to be watered, whereas the drought group plants received no water for 3 d. On the fourth day of drought stress, plant material was harvested in liquid nitrogen and transferred to a −80 °C freezer. Fresh leaves were used for RWC estimation. The estimation of RWC was done following the formula:


RWC(%)=[(FW−DW)/(TW−DW)]×100,

where FW is fresh weight, TW is the turgid weight of leaf samples kept in deionized water for 24 h, and DW is the dry weight of leaf samples after drying at 65 °C in a hot air oven until a constant dry weight is achieved.

### Determination of sulfite, sulfide, cysteine, sulfate, and hydrogen peroxide

Sulfite, sulfide, and L-cysteine in leaf samples were extracted (1:4, w/v) in a 0.1 M Tris–HCl, pH 8.0 buffer containing 0.5 M sodium sulfate. The extract was centrifuged (18 000*×g*, 15 min) and de-proteinized using a Sephadex G-25 Column (Pd-10, GE Healthcare, Sweden). For sulfide, 600 µl of 2% cadmium acetate was added to the 600 µl of the eluate and kept on ice immediately after the separation through the column. The sulfide concentration was detected using 40 µl of 4 mM *N*-dimethyl-1,4-phenylenediammonium-dichloride dissolved in 7.2 M HCl and 4 µl 0.6 mM FeCl_3_ dissolved in 1.2 M HCl, detected colorimetrically at 625 nm (Kurmanbayeva *et al*., 2022). The concentration of sulfite in the eluate was detected by employing a chicken sulfite oxidase kit, according to the manufacturer's instructions (Roche, Germany; Brychkova *et al*., 2013; Bekturova *et al*., 2021). Cysteine was detected with 10% trichloroacetic acid, acetic acid, and ninhydrin at 560 nm (Kurmanbayeva *et al*., 2022).

Sulfate concentration was determined by extracting 20 mg of frozen leaves in 1 ml of ultrapure Milli-Q water. Extracts were centrifuged for 10 min at 21 000*×g* at 4 °C twice, and then the supernatant was diluted 1:4 (v/v) with Milli-Q water. The obtained extracts were filtered (0.22 μm, Millipore), and ion chromatography was used to determine the sulfate concentration using a Dionex IonPac AS19 662 4×250 mm column. H_2_O_2_ content in extracted leaves was determined using a colorimetric assay based on 3,5-dichloro-2-hydroxobenzene sulfonate, which couples oxidatively to 4-aminoantipyrine in the presence of H_2_O_2_ and horseradish peroxidase to yield a red quinoneimine dye (Fossati *et al*., 1980).

### Detection of abscisic acid and its derivatives

ABA and its derivatives, namely phaseic acid (PA), neophaseic acid (neoPA), ABA-glucose ester (ABAGE), and 7′-hydroxy-ABA (7′-OH-ABA), were detected as described in Turečková *et al*. (2009). Briefly, 100 mg of leaf tissue was freeze-dried and extracted in 0.75 ml ice-cold methanol–water–acetic acid (10/89/1, v/v) with sodium diethyldithiocarbamate and internal standards (20 pmol each of d2-ABA alcohol, d2-ABA aldehyde, d3-PA, d3-DPA, d3-neoPA, d4-7′-OH-ABA, d5-ABAGE, and d6-(+)ABA) for 1 h. After 1 h, samples were centrifuged at 18 000*×g* for 10 min at 4 °C, and the supernatant was transferred to a separate tube. The pellet was resuspended, and the above step was repeated for extraction. The supernatant was transferred onto 3 ml Oasis® HLB cartridges conditioned with 2 ml of methanol and equilibrated with 2 ml of methanol–water–acetic acid (10/89/1, v/v), washed with 1 ml of methanol–water–acetic acid (10/89/1, v/v) and eluted with 3 ml of methanol–water–acetic acid (80/19/1, v/v). The eluates were dried under a vacuum, and a purified Acquity UPLC System (Waters, Milford, MA, USA), combined with a Micromass Quattro micro API mass spectrometer (Waters MS Technologies, Manchester, UK), was used to detect ABA and its derivatives. The QuanLynx module did data processing within the MassLynx software (version 4.1, Waters, Milford, MA, USA).

### Determination of protein expression

As described, soluble proteins were extracted from Arabidopsis rosettes and tomato leaves (Sagi *et al*., 1998). Concentrations of total soluble protein in the resulting supernatant were determined according to Bradford (1976). A western blot was analysed as previously described (Brychkova *et al*., 2013). Briefly, 10 μg of crude protein extract was electrophoresed using SDS-PAGE, transferred to a polyvinylidene fluoride (PVDF) membrane, blocked, and thereafter incubated with specific primary antibodies [APR2 (gift of Prof. S. Kopriva, University of Cologne, Germany), SiR (kindly provided by S. Heinhorst, University of Southern Mississippi, USA), and SO (primary antibodies raised against the SO synthetic polypeptide, RHPSLKINAKEPFNAE)]. Primary antibodies of SO were diluted 500-fold in phosphate buffered saline (PBS), and secondary antibodies (anti-guinea pig IgG, Sigma) were diluted 10 000-fold in PBS. The SiR primary antibody was diluted 5000-fold in PBS, the APR antibody was diluted 1000-fold, and the secondary antibody (anti-rabbit IgG, Sigma) was diluted 12 000-fold in PBS. The PVDF membranes were scanned using the Gel-Doc system (ChemiDoc Touch imaging system, Bio-Rad Laboratories), and the intensity was measured with ImageJ software.

### Aldehyde oxidase activity

Crude protein was extracted as previously described and fractionated on a discontinuous non-denaturing 7.5% (w/v) polyacrylamide gel (Laemmli, 1970) at 4 °C. After fractionation, the gel was incubated in 50 mM Tris–HCl, pH 7.5, containing 1 mM 3-(4,5-dimethyl-2-thiazolyl)-2,5-diphenyl-2*H*-tetrazolium bromide (MTT) and 0.1 mM *N*-methylphenazonium methyl sulfate (PMS) with an abscisic aldehyde substrate for 30 min. Deriving from aldehyde oxidase activity, the final product, formazan, was scanned with the Gel Doc system, and the activity band intensity was measured with ImageJ software.

### Preparation of RNA and quantitative real time-PCR

For quantitative RT–PCR, total RNA was extracted from Arabidopsis and tomato leaves, using a plant/fungi total RNA Purification Kit according to the manufacturer's instructions (Norgen Biotek Corp., Canada). Arabidopsis or tomato total RNA (0.5 μg) was reverse-transcribed with an iScript cDNA Synthesis Kit (Bio-Rad Laboratories). Details of primers and PCR conditions are given in Supplementary Table S3. Quantitative RT-PCR reactions contained 1:10 v/v first-strand cDNA as the template, specific primers, and iQ™ SYBR® Green Master Super Mix (Bio-Rad Laboratories). Fluorescence increments of each reaction were simultaneously monitored using the CFX Connect Real-Time PCR Detection System (Bio-Rad Laboratories). The quantitative analysis of the transcripts was performed as previously described (Brychkova *et al*., 2007, 2013; Yarmolinsky *et al*., 2013). Relative expression was calculated according to Livak and Schmittgen (2001). Reference gene suitability was confirmed using the two one-sided tests (TOST) equivalence procedure (Lakens, 2017; Curis *et al*., 2019) with bounds of (−0.5, +0.5) *C*_q_ (2^0.5^=1.414-fold change at 100% PCR efficiency) as shown in Supplementary Table S4. Collectively, these metrics demonstrate that *elongation factor 1-α* (*EF1-α*) exhibits stable expression across genotypes, treatments, and independent experiments, supporting its suitability as a reference gene for normalization under the tested conditions, in line with MIQE recommendations (Bustin *et al*., 2009, 2025). *EF1-α* is commonly used as a reference gene in RT-qPCR studies in Arabidopsis and tomato, where its use in combination with other housekeeping genes has been shown to yield consistent expression patterns (Gong *et al*., 2010; Dekkers *et al*., 2012; Yarmolinsky *et al*., 2013; Li *et al*., 2015; Egea *et al*., 2018; Bekturova *et al*., 2021; Tapia *et al*., 2021), supporting its selection for normalization in the present study. The expression of genes in each treated genotype was compared with the WT (Col-0) for Arabidopsis and WT (RR) for tomato under control conditions and presented as relative expression.

### Statistical analysis

Statistical analyses were conducted using IBM SPSS Statistics 26 (IBM Corp., Armonk, NY, USA) and GraphPad Prism 10 (GraphPad Software, Boston, MA, USA). Two-way analysis of variance (ANOVA) followed by Tukey’s multiple comparison test was applied to determine statistically significant differences. Graphs were generated using GraphPad Prism 10. Images obtained from the in-gel documentation system (ChemiDoc Touch Imaging System; Bio-Rad Laboratories) were processed using Image Lab software version 5.2.1 (Bio-Rad Laboratories). Relative intensities from in-gel electrophoretic activity assays and western blot analyses were quantified using ImageJ software.

## Results

### Sulfite oxidase deficiency resulted in the highest water loss under drought stress, compared with wild-type and selected Arabidopsis mutants of the sulfate reduction pathway

Recent findings demonstrated that altered sulfite homeostasis led to higher water loss in *SO* Ri mutants than in WT Arabidopsis plants after sulfite infiltration into rosette leaves or plant detachment from roots. Additionally, a brief irrigation cessation (for 48 h) in Arabidopsis plants, grown in a limited volume of field-capacity-watered low-nutrient soil, can induce a higher water loss in *SO* Ri than in WT and *SO* OE Arabidopsis mutants (Bekturova *et al*., 2021). However, how sulfite homeostasis occurs in Arabidopsis plants under an actual drought onset has not yet been studied. In our study, 23-day-old WT, *SO* OE, *SO* Ri, *apr2* KO, and *apr2* KO*/SO* OE plants were irrigated to the soil’s field-capacity level (defined after excess water removal by gravity). Then, irrigation was stopped for up to 6 d to observe the phenotypes at the onset of drought. On the sixth day of drought stress, the *SO* Ri mutant showed the most wilting symptoms and damage, followed by the WT plants, which showed wilting of several leaves (Fig. 1A). In contrast, the *SO* OE, *apr2* KO, and *apr2* KO/*SO* OE mutants did not show any wilting or damage symptoms. These results suggest that the *SO* Ri mutant has the highest susceptibility to drought stress, followed by WT, with the *SO* OE, *apr2* KO, and *apr2* KO/*SO* OE mutants having a relatively higher tolerance (Fig. 1A).

**Fig. 1.**
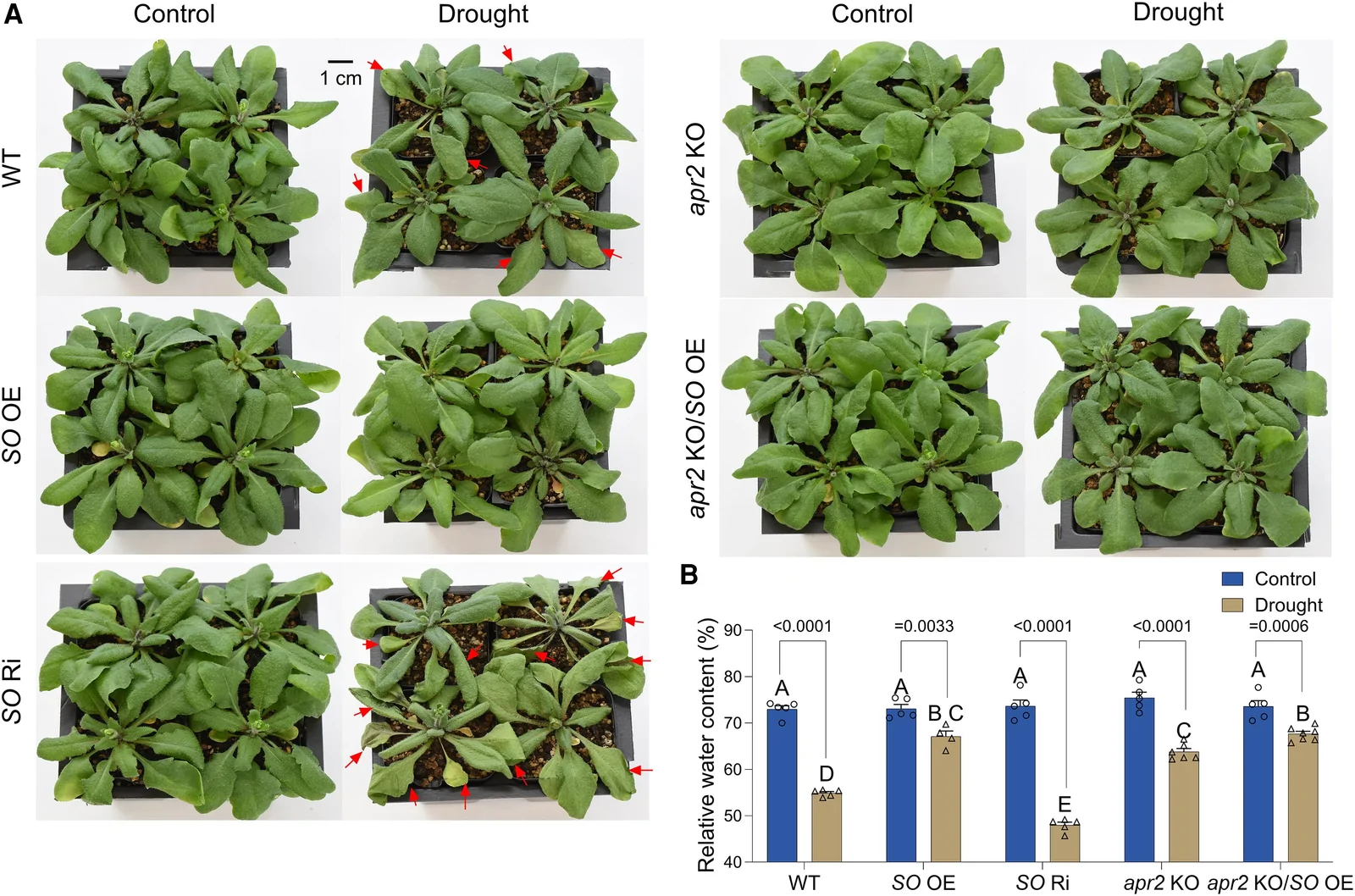
Effects of drought stress on water status in Arabidopsis wild type (WT), *sulfite oxidase* overexpression (*SO* OE), *SO* RNA interference (*SO* Ri), *5′-adenylylphosphosulfate reductase 2* knockout (*apr2* KO), and *apr2* KO/*SO* OE leaves. (A) Phenotype of the WT, *SO* OE, *SO* Ri, *apr2* KO, and *apr2* KO/*SO* OE plants under control and drought conditions on the sixth day of treatment. (B) The relative water content of the control and drought leaves measured on the sixth day of treatment. Two-way ANOVA revealed significant main effects of genotype (*P*<0.0001), treatment (*P*<0.0001), and a significant genotype×treatment interaction (*P*<0.0001), followed by Tukey's multiple comparison test for pairwise comparisons. Different uppercase letters indicate significant differences among genotypes regardless of treatment (*n*=5) (red arrow indicates wilting).

Following the wilting, the *SO* Ri mutants displayed significantly lower RWC, under drought stress, than the WT plants (Fig. 1B). In contrast, the significantly higher RWC in the *SO* OE, *apr2* KO, and *apr2* KO/*SO* OE plants than in the WT and *SO* Ri plants indicated these mutants enhanced drought tolerance capacity. These results demonstrate the highest susceptibility of *SO* Ri plants to drought stress among the genotypes tested.

### Sulfite homeostasis orchestrated by sulfite oxidase, adenosine-5′-phosphosulfate reductase 2, and sulfite reductase plays a significant role in Arabidopsis drought tolerance

Under control conditions, sulfite levels were comparable among all genotypes. The sulfite level was significantly increased in the *SO* Ri mutants under drought stress compared with the *SO* OE, *apr2* KO, and *apr2* KO/*SO* OE plants (Fig. 2A). The accumulated sulfite can be attributed to the decrease in SO and SiR protein expressions in the *SO* Ri mutant under drought. In contrast, the sulfite level did not change in the *SO* OE, *apr2* KO and *apr2* KO/*SO* OE mutants under drought stress, due to increased SO and decreased APR protein expression (Fig. 2B, C). These results show a direct correlation between enhanced sulfite accumulation and increased water loss in *SO* Ri plants under drought stress, as the result of SO, SiR, and APR2 expression levels in Arabidopsis mutants.

**Fig. 2.**
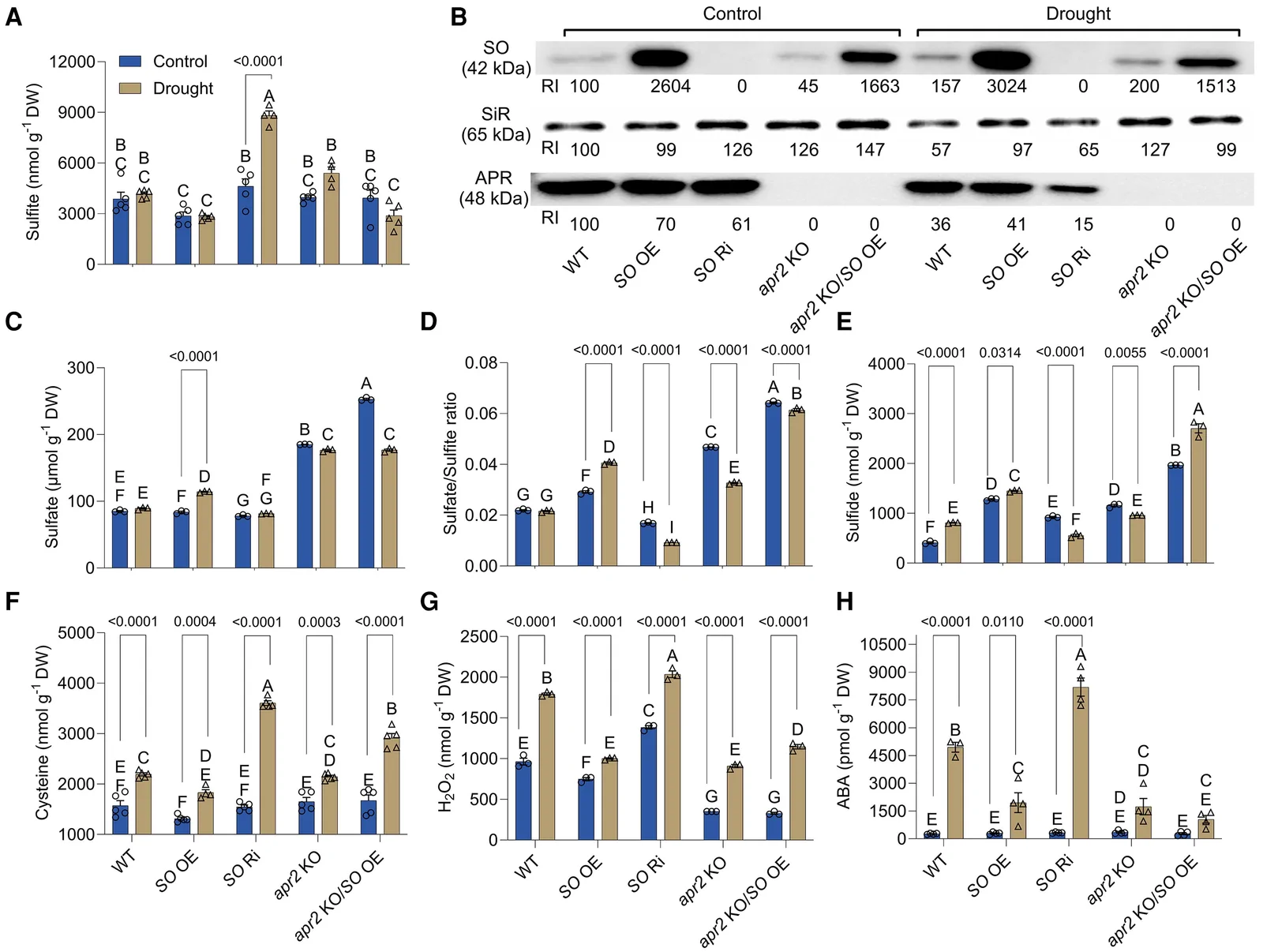
Effects of drought stress on metabolites and protein expression in Arabidopsis wild type (WT), *sulfite oxidase* overexpression (*SO* OE), *SO* RNA interference (*SO* Ri), *5′-adenylylphosphosulfate reductase 2* knockout (*apr2* KO), and *apr2* KO/*SO* OE leaves. (A) Sulfite content [nmol g^−1^ dry weight (DW)]. (B) SO, sulfite reductase (SiR), and APR protein accumulation detected by western blotting with recombinant antibodies against SO, SiR, and APR. (C) Sulfate content (µmol g^−1^ DW). (D) Sulfate/sulfite ratio. (E) Sulfide content (nmol g^−1^ DW). (F) Cysteine content (nmol g^−1^ DW). (G) H_2_O_2_ (nmol g^−1^ DW). (H) Abscisic acid (ABA) content (pmol g^−1^ DW). Values are for control and drought leaves measured on the sixth day of treatment. RI, relative intensity. Two-way ANOVA revealed significant main effects of genotype (*P*<0.0001), treatment (*P*<0.0001), and a significant genotype×treatment interaction (*P*<0.0001), followed by Tukey's multiple comparison test for pairwise comparisons. Different uppercase letters indicate significant differences among genotypes regardless of treatment (*n*=4).

It was previously shown that enhanced sulfate levels induced stomatal closure and reduced water loss in Arabidopsis plants (Batool *et al*., 2018). The current study detected the lowest sulfate content in the *SO* Ri mutants under both control and drought conditions, compared with the other Arabidopsis lines examined, due to the reduced SO protein expression. In contrast, the *apr2* KO and *apr2* KO/*SO* OE lines showed significantly higher sulfate levels than other Arabidopsis mutants and WT plants under control and drought conditions. As expected, the sulfate content was markedly increased in the *SO* OE mutants under drought stress compared with the control plants (Fig. 2C).

The results indicate that the combination of sulfate and sulfite levels affects plant water loss, rather than each acting as a single factor. Indeed, estimation of the sulfate/sulfite ratio was correlated with water loss in the Arabidopsis WT and its mutants (Fig. 2D; numeric sulfate in µmol/numeric sulfite in nmol). Significantly less water loss and fewer drought-tolerant phenotypes in the *SO* OE, *apr2* KO, and *apr2* KO*/SO* OE mutants were correlated with a higher sulfate/sulfite ratio under drought stress conditions than in the WT plants. In contrast, the *SO* Ri mutants showed a decreased sulfate/sulfite ratio compared with the WT plants under drought stress, which correlates with their higher water loss and susceptibility to drought stress. Further, the sulfate/sulfite ratio significantly decreased under drought stress in the *SO* Ri mutants compared with the control, supporting the finding of their higher water loss under drought stress. As in the *SO* Ri, the sulfate/sulfite ratio significantly decreased in the *apr2* KO mutants under drought stress compared with the control. However, while the *apr2* KO mutant exhibited significantly increased sulfite in response to drought, compared with the control, its considerably higher sulfate/sulfite ratio than in the WT plants (under both control and drought stress conditions) resulted in less water loss in this mutant.

H_2_S acts as a signaling molecule regulating ABA-mediated stomatal movement (Chen *et al*., 2020). The sulfide content was significantly higher in the *SO* overexpressing lines (*SO* OE and *apr2* KO*/SO* OE) than in the WT plants under both control and drought conditions (Fig. 2E). In contrast, the *SO* Ri mutants showed significantly lower sulfide than the WT plants under drought conditions, likely the result of the reduced SiR protein (which catalyses sulfite to sulfide) under drought stress (Fig. 2B).

Previous research suggests sulfate might integrate into cysteine, promoting ABA biosynthesis by activating Arabidopsis aldehyde oxidase 3 (AAO3; Batool *et al*., 2018). In our study, the cysteine concentration increased in all the Arabidopsis mutants under drought stress (Fig. 2F). Surprisingly, the *SO* Ri mutants showed the highest cysteine level under drought stress, compared with the other mutants. However, the decrease in sulfide, an essential substrate for L-cysteine biosynthesis, suggests lowered L-cysteine biosynthesis in the *SO* Ri mutants. Accordingly, the significantly lower total protein in the *SO* Ri mutants than in the WT plants, under drought stress, suggests higher L-cysteine content due to increased protein degradation rather than cysteine biosynthesis (Supplementary Fig. S1A). Previous studies have confirmed that protein degradation can increase L-cysteine concentrations (Slavikova *et al*., 2008; Brychkova *et al*., 2013).

### Elevated sulfite in *sulfite oxidase* RNA interference plants suppresses abscisic acid signal transduction despite abscisic acid accumulation, accounting for the lowest relative water content among genotypes tested

Sulfite accumulation was shown to induce reactive oxygen species generation by NADPH oxidase and other FAD-containing enzymes in plant leaves (Brychkova *et al*., 2012a; Yarmolinsky *et al*., 2014; Bekturova *et al*., 2021). Additionally, H_2_O_2_ enhancement in leaf stomata and toxic aldehydes in leaves have been demonstrated in response to increased sulfite (Bekturova *et al*., 2021). Accordingly, the H_2_O_2_ concentration significantly increased in all the Arabidopsis plants exposed to drought stress, compared with the unstressed control plants (Fig. 2G). Significantly, the H_2_O_2_ concentration was inversely correlated with the sulfate/sulfite ratio (compare Fig. 2G with Fig. 2D); the *SO* OE, *apr2* KO, and *apr2* KO*/SO* OE mutants exhibited significantly lower H_2_O_2_ levels than the WT plants, while the *SO* Ri mutants showed a significantly higher H_2_O_2_ level than the WT plants under drought stress. The observed enhancement of H_2_O_2_ concentrations, alongside sulfite increase, accompanied by a RWC decrease in the plant leaves, indicates that the changes in H_2_O_2_ concentration in response to drought stress are not related to its known role in ABA-induced stomatal closure and decreased water loss (Bekturova *et al*., 2021).

ABA is crucial in plant drought stress response and water loss via stomatal aperture control (Lim *et al*., 2015). Notably, no significant difference in ABA levels was observed among the Arabidopsis plants under control conditions (Fig. 2H). However, under drought stress, the *SO* Ri mutants exhibited significantly higher ABA levels than the WT plants, indicating a higher drought stress intensity. In contrast, significantly lower ABA levels were detected in the *SO* OE, *apr2* KO, and *apr2* KO*/SO* OE mutants, compared with the WT leaves, indicating that these plants were less affected by drought. In support of the observed elevation of ABA levels in the *SO* Ri and WT, simultaneously higher levels of inactive ABA metabolites, such as phaseic acid, neophaseic acid, ABA glucose ester, and (+)7-OH-ABA, were seen. Compared with the *SO* OE, *apr2* KO, and *apr2* KO/*SO* OE mutants, these elevated ABA degradation products in both the WT and *SO* Ri plants are consistent with less ABA in the latter genotypes exposed to drought stress (Supplementary Fig. S1).

To further investigate the molecular basis of impaired stomatal closure in *SO* Ri plants, the relative expression of key ABA downstream signaling and stomatal movement genes was examined by qRT-PCR in WT, *SO* OE, *SO* Ri, *apr2* KO, and *apr2* KO/*SO* OE plants under control and drought stress conditions (Supplementary Fig. S2). Under control conditions, no significant differences were observed in *Pyrobactin resistance 1-like 5* (*PYL5*) expression among genotypes (Supplementary Fig. S2A). Under drought, genotype-specific differences had emerged. *PYL5* (Supplementary Fig. S2A) was most severely suppressed in *SO* Ri, falling to the absolute lowest value of all genotypes and conditions, well below both its own irrigated control and WT under drought, indicating drought-specific suppression of ABA receptor expression in *SO* Ri. *Highly ABA-induced PP2C gene 2* (*HAI2*), a PP2C phosphatase and negative regulator of ABA signaling, was induced by drought in all genotypes relative to their irrigated controls (Supplementary Fig. S2B). *HAI2* expression was highest in *SO* Ri, followed by *apr2* KO, WT, *SO* OE, and *apr2* KO/*SO* OE under drought conditions. The strong *HAI2* induction in *SO* Ri relative to WT and the tolerant genotypes indicates that *SO* Ri, despite accumulating the most ABA, simultaneously suppresses ABA perception (low PYL5) and activates the strongest ABA negative feedback (high HAI2), contributing to the paradox of high ABA with impaired water retention. *Guard cell hydrogen peroxide-resistant 1* (*GHR1*) expression was low and not drought induced in *SO* Ri and WT, while *SO* OE and *apr2* KO/*SO* OE showed higher GHR1 under drought (Supplementary Fig. S2C). *SLAC1 homologue 2* was actively suppressed in *SO* Ri under drought conditions relative to its irrigated control, while *apr2* KO/*SO* OE maintained higher expression (Supplementary Fig. S2D). Ca^2+^-ATPase expression was significantly lower in *SO* Ri compared with WT and all genotypes under drought condition (Supplementary Fig. S2E). The Hyperpolarization-activated inward-rectifying potassium channel *KAT1* underwent the most dramatic suppression in *SO* Ri under drought, falling to the absolute lowest value of all genotypes, well below its own irrigated control and WT under drought (Supplementary Fig. S2F). Collectively, these data showed that *SO* Ri under drought is characterized by suppressed ABA perception, the strongest ABA negative feedback, and reduced expression of key guard cell ion transport components relative to its irrigated control, consistent with most likely impaired stomatal regulation. These gene expression differences are secondary to the primary sulfite and sulfate/sulfite ratio as the key determinants of water retention; notably, *HAI2* was the only gene substantially induced in WT under drought, suggesting that HAI2-mediated suppression of ABA signaling may play an important role in limiting the effectiveness of drought-induced ABA accumulation in both WT and *SO* Ri plants.

### Overexpression of sulfite oxidase and sulfite reductase improved sulfite detoxification and increased drought tolerance in Arabidopsis

Overexpression of the tomato (*Solanum lycopersicum*) *SiR* in the Arabidopsis *SO* OE mutant (*SlSiR* OE/*SO* OE) and the mutants generated by using CRISPR-Cas9 genome editing (multiplexed CRISPR–Act 3.0 system; Pan *et al*., 2021), the Arabidopsis *SiR* and *SO* in a Col-0 background (*SiR* OE*^C2^*+*SO* OE*^C2^*) and the Arabidopsis *SiR* in an *SO* OE background (*SiR* OE*^C4^*/*SO* OE), were used to examine further whether *SO* overexpression, together with *SiR* overexpression, improves plant resistance to drought. Indeed, the above-mentioned double mutants overexpressing both *SO* and *SiR* exhibited improved drought stress resilience compared with the WT plants (Supplementary Fig. S3). Overexpression of *SO* and *SiR* transcripts and proteins in these mutants was confirmed by qPCR (Supplementary Fig. S4A, B; employing primers for Arabidopsis *SiR* and *SO* shown in Supplementary Table S3) and western blotting (Fig. 3A). On the sixth day of irrigation cessation, the WT plants started wilting, whereas the *SlSiR* OE/*SO* OE, *SiR* OE*^C2^*+*SO* OE*^C2^*, and *SiR* OE*^C4^*/*SO* OE mutants did not show any wilting symptoms (Supplementary Fig. S3). The RWC of all the plant genotypes was significantly reduced under drought stress (Fig. 3B). However, the WT plants exhibited significantly lower RWC than the *SlSiR* OE/*SO* OE, *SiR* OE*^C2^*+*SO* OE*^C2^*, and *SiR* OE*^C4^*/*SO* OE mutants exposed to drought stress.

**Fig. 3.**
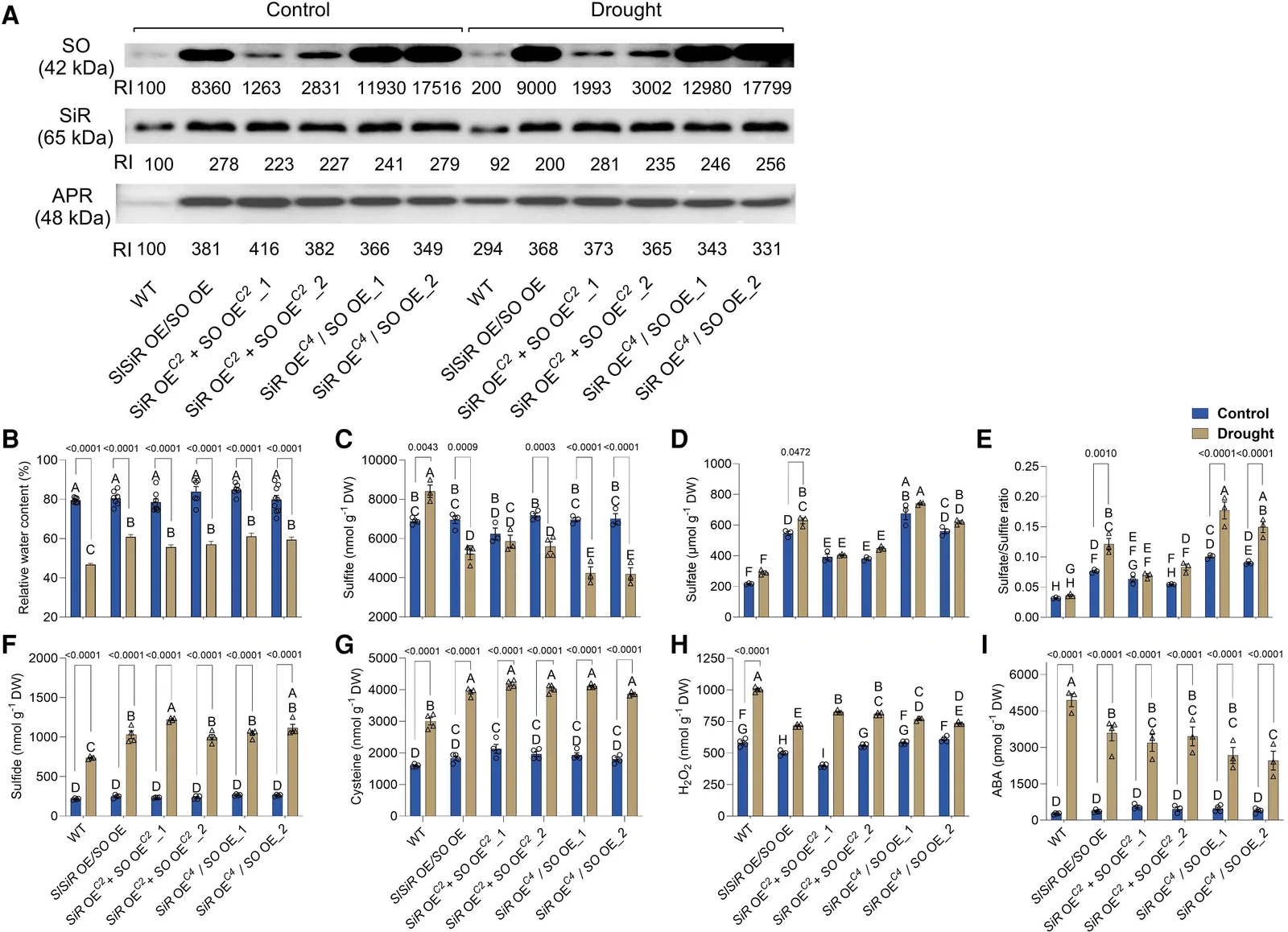
Effects of drought stress on metabolites and protein expression in Arabidopsis wild type (WT), *Solanum lycopersicum sulfite reductase* (*SiR*) in an Arabidopsis *sulfite oxidase* (*SO*) OE background (*SlSiR* OE/*SO* OE), Arabidopsis *SiR* and *SO* in a Col-0 background (*SiR* OE*^C2^*+*SO* OE*^C2^*) and Arabidopsis *SiR* in an *SO* OE background (*SiR* OE*^C4^*/*SO* OE). (A) SO, SiR, and adenosine-5′-phosphosulfate reductase (APR) protein accumulation (RI, relative intensity). (B) Relative water content (RWC, %). (C) Sulfite content [nmol g^−1^ dry weight (DW)]. (D) Sulfate content (µmol g^−1^ DW). (E) Sulfate/sulfite ratio. (F) Sulfide content (nmol g^−1^ DW). (G) L-Cysteine content (nmol g^−1^ DW). (H) H_2_O_2_ content (nmol g^−1^ DW). (I) Abscisic acid (ABA) content (pmol g^−1^ DW). Values are for control and drought leaves measured on the sixth day of treatment. Two-way ANOVA followed by Tukey's multiple comparison test was applied to all panels. Significant main effects of genotype and treatment (both *P*<0.0001) were detected across all panels. Genotype×treatment interaction *P*-values were as follows: RWC (*P*=0.0002), sulfite (*P*<0.0001), sulfate (*P*=0.3582), sulfate/sulfite ratio (*P*<0.0001), sulfide (*P*<0.0001), L-cysteine (*P*<0.0001), H_2_O_2_ (*P*<0.0001), ABA (*P*<0.0001). Different uppercase letters indicate significant differences among genotypes regardless of treatment (*n*=5). OE*^C2^* and OE*^C4^*: *C* indicates CRISPR, and 2 and 4 indicate the number of guide RNA.

To examine whether, similar to the WT, *SO* OE, *SO* Ri, *apr2* KO, and *apr2* KO/*SO* OE mutants, the resistance to drought stress is related to sulfur-containing metabolites (Fig. 2), the S-containing metabolites were detected in the WT and *SlSiR* OE/*SO* OE, *SiR* OE*^C2^*+*SO* OE*^C2^*, and *SiR* OE*^C4^*/*SO* OE mutants. Under control conditions, the sulfite content in leaves was similar in all Arabidopsis plants (Fig. 3C). However, the sulfite content was significantly higher in the WT plants than in the *SlSiR* OE/*SO* OE, *SiR* OE*^C2^*+*SO* OE*^C2^*, and *SiR* OE*^C4^*/*SO* OE mutants under drought stress. Significantly, the lower sulfite content in the *SlSiR* OE/*SO* OE, *SiR* OE*^C2^*+*SO* OE*^C2^*, and *SiR* OE*^C4^*/*SO* OE mutants, under drought, can most likely be attributed to the increased oxidation of sulfite to sulfate and the reduction to sulfide caused by overexpressing *SO* and *SiR*, respectively. The protein expression of APR2 was higher in Arabidopsis *SlSiR* OE/*SO* OE, *SiR* OE^C2^+*SO* OE^C2^, and *SiR* OE^C4^/*SO* OE plants compared with WT under control and drought stress (Fig. 3A). Accordingly, sulfate levels were significantly higher in the leaves of the *SlSiR* OE/*SO* OE, *SiR* OE*^C2^*+*SO* OE*^C2^*, and *SiR* OE*^C4^*/*SO* OE mutants than in the WT leaves, under both control and drought stress conditions (Fig. 3D).

The sulfate/sulfite ratio was significantly higher in the *SlSiR* OE/*SO* OE, both *SiR* OE*^C4^*/*SO* OE mutants examined, and one of the *SiR* OE*^C2^*+*SO* OE*^C2^* mutants tested (while in the second one, the ratio increased insignificantly) than in the WT plants, under both control and drought stress conditions (Fig. 3E). Similar to the previous experiment employing the different examined mutants (Figs 1B, 2D), the increased sulfate/sulfite ratio showed a clear correlation with increased drought stress resistance and improved RWC in the *SlSiR* OE/*SO* OE, *SiR* OE*^C2^*+*SO* OE*^C2^*, and *SiR* OE*^C4^*/*SO* OE mutants. Due to *SiR* overexpression, the sulfide and cysteine contents in the leaves were significantly higher in the *SlSiR* OE/*SO* OE, *SiR* OE*^C2^*+*SO* OE*^C2^*, and *SiR* OE*^C4^*/*SO* OE mutants than in the WT under drought stress (Fig. 3F, G). In contrast, the hydrogen peroxide and ABA levels were significantly lower in the *SlSiR* OE/*SO* OE, *SiR* OE*^C2^*+*SO* OE*^C2^*, and *SiR* OE*^C4^*/*SO* OE mutants than in the WT plants under drought stress conditions (Fig. 3H, I), supporting the finding of less water loss in the mutant plants, as previously explained. Supporting the validity of the observed lower ABA levels in the mutant leaves, compared with the WT leaves, the simultaneously inactive ABA degradation products—including phaseic acid, neophaseic acid, ABA glucose ester, and (+)7-OH-ABA—were significantly reduced in *SlSiR* OE/*SO* OE, *SiR* OE*^C2^*+*SO* OE*^C2^*, and *SiR* OE*^C4^*/*SO* OE mutants compared with WT leaves, under the drought stress conditions (Supplementary Fig. S4C–F). Significantly, the lower transcript expression of NCED3, the committed step of ABA biosynthesis (Ruggiero *et al*., 2004; Sussmilch *et al*., 2017), and AAO3, which is responsible for the last step in ABA biosynthesis (Sagi *et al*., 1998, 1999 and references therein), is also consistent with the reduced ABA content in the *SlSiR* OE/*SO* OE, *SiR* OE*^C2^*+*SO* OE*^C2^*, and *SiR* OE*^C4^*/*SO* OE mutants, compared with the WT plants, under the drought stress conditions (Supplementary Fig. S4G, H). Overall, these results suggest that overexpression of *SO* and *SiR* improved sulfite detoxification and increased drought tolerance in Arabidopsis in the *SlSiR* OE/*SO* OE, *SiR* OE*^C2^*+*SO* OE*^C2^*, and *SiR* OE*^C4^*/*SO* OE mutants compared with the WT plants.

### *Sulfite oxidase* and *sulfite reductase* overexpression improved sulfite detoxification and drought tolerance in tomato plants

The results in Arabidopsis led us to investigate the role of *SiR* OE and *SO* OE in drought stress resilience in a crop plant. The tomato wild type [*Solanum lycopersicum* ‘Rheinlands Ruhm’ (RR)], *SO* OE, *SiR* OE, and the double mutant *SiR* OE/*SO* OE in the RR background were employed. The overexpression of *SO* and *SiR* that we previously used (Brychkova *et al*., 2007; Yarmolinsky *et al*., 2013) and the generated double mutant were confirmed by qPCR and western blotting (Supplementary Fig. S5). Thirty-day-old tomato plants, grown in pots filled with 25 g of low-nutrient soil, were exposed to an irrigation cessation (drought stress) for 4 d. In contrast, plants of similar genotypes were exposed to control conditions with regular water supply every other day. A similar phenotype was observed in all tomato plants grown under control conditions (Fig. 4, upper line of plants). However, under drought stress, the RR plants showed clearly visible wilting symptoms compared with the other tomato genotypes studied (Fig. 4, RR in the lower line of plants). The RWC level supported the visible phenotype, as it was significantly reduced in all tomato genotypes under drought stress compared with the control plants (Fig. 5A). The *SO* OE, *SiR* OE, and *SiR* OE/*SO* OE tomato plants showed significantly higher RWC than the RR plants under drought stress. These results indicate that overexpression of *SO* in *SO* OE, *SiR* in *SiR* OE, and both in *SiR* OE/*SO* OE tomato plants improved drought stress resilience and made them less susceptible to drought stress than the wild-type RR plants.

**Fig. 4.**
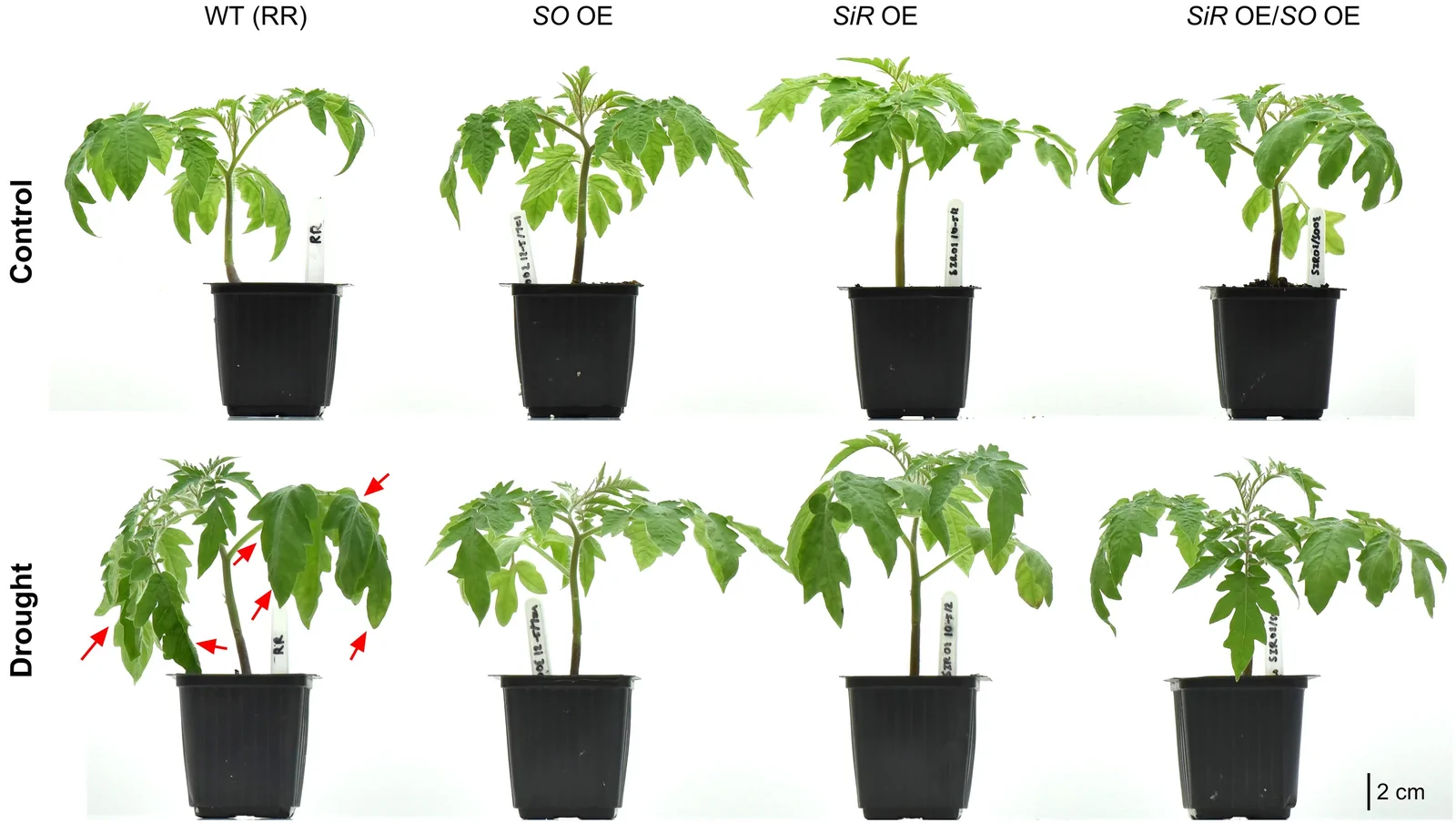
Phenotype of tomato Rheinlands Ruhm (RR; wild type), *sulfite oxidase* overexpression (*SO* OE), *sulfite reductase* overexpression (*SiR* OE), and *SiR* OE/*SO* OE double overexpression mutant plants under control and drought conditions on the fourth day (red arrow indicates wilting).

**Fig. 5.**
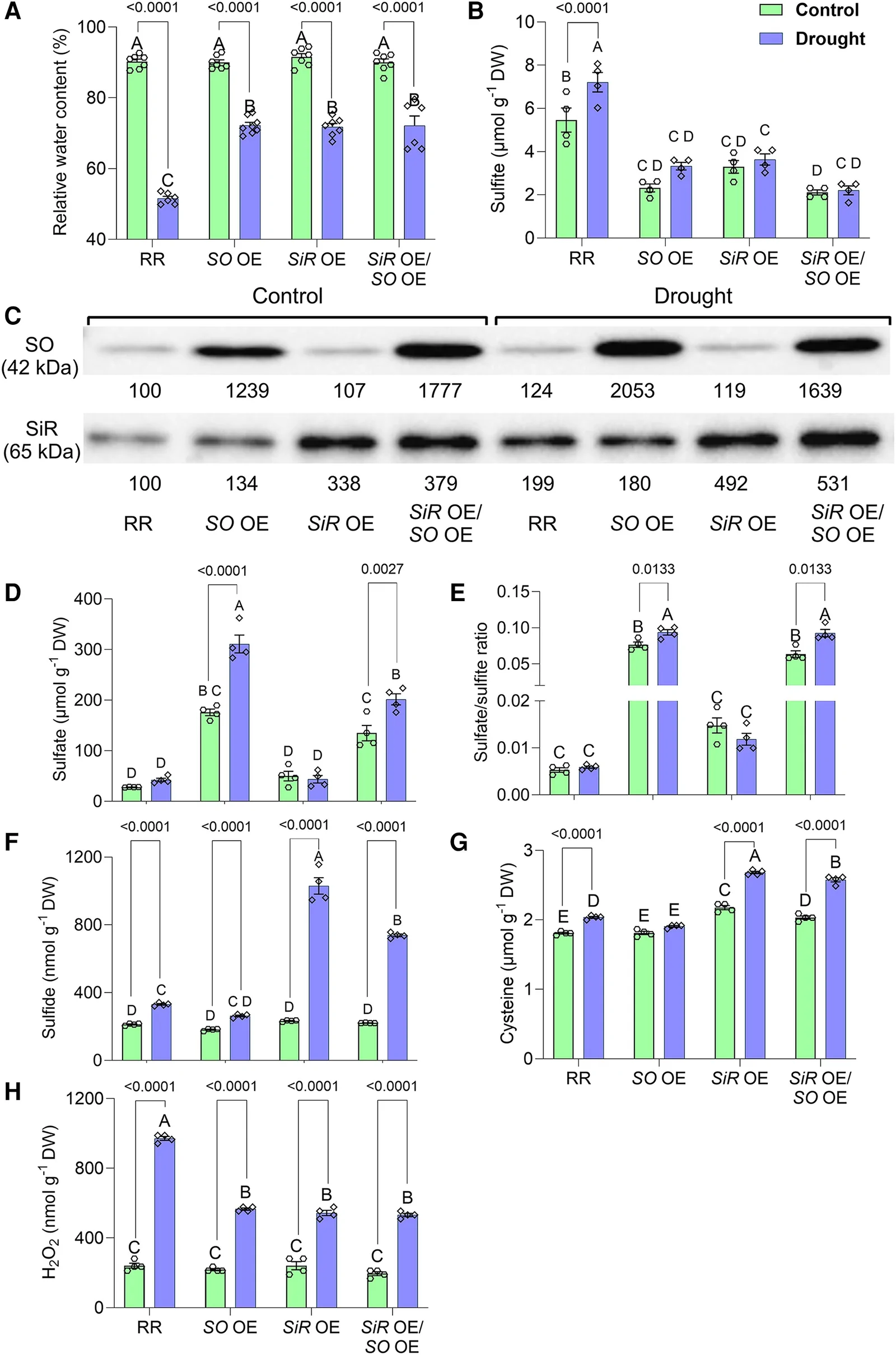
Effects of drought stress on water status, protein expression, and metabolites of the tomato plant in Rheinlands Ruhm (RR; wild type), *sulfite oxidase* overexpression (*SO* OE), *sulfite reductase* overexpression (*SiR* OE), *SiR* OE and *SO* OE double overexpression (*SiR* OE/*SO* OE) plants. (A) Relative water content (RWC) of control (*n*=7) and drought (*n*=8) leaves. (B) Sulfite [µmol g^−1^ dry weight (DW)]. (C) SO and SiR protein accumulation (RI, relative intensity). (D) Sulfate (µmol g^−1^ DW). (E) Sulfate/sulfite ratio. (F) Sulfide (nmol g^−1^ DW). (G) L-Cysteine (µmol g^−1^ DW). (H) H_2_O_2_ content (nmol g^−1^ DW). Content of control and drought leaves measured on the fourth day of treatment. Two-way ANOVA followed by Tukey's multiple comparison test was applied to all panels. Significant main effects of genotype and treatment (both *P*<0.0001) were detected across all panels. Genotype×treatment interaction *P*-values were as follows: RWC (*P*<0.0001), sulfite (*P*=0.0604), sulfate (*P*<0.0001), sulfate/sulfite ratio (*P*<0.0001), sulfide (*P*<0.0001), cysteine (*P*<0.0001), H_2_O_2_ (*P*<0.0001). Different uppercase letters indicate significant differences among genotypes regardless of treatment (*n*=4).

The sulfite level was significantly lower in the *SO* OE, *SiR* OE, and *SiR* OE/*SO* OE tomato plants than in the RR plants under both control and drought stress (Fig. 5B). These lower sulfite levels may result from the overexpressed SO oxidizing sulfite to sulfate and the overexpressed SiR protein reducing sulfite to sulfide (Fig. 5C). Indeed, the sulfate content was considerably increased in the *SO* OE and *SiR* OE/*SO* OE plants under both control and drought stress conditions, compared with the RR plants (Fig. 5D). Similarly, the sulfate/sulfite ratio was significantly higher in the *SO* OE and *SiR* OE/*SO* OE tomato plants in response to drought stress, while the *SiR* OE plants, which were not affected by the drought, still had a much higher sulfate/sulfite ratio than the RR plants under both control and drought stress conditions, supporting the finding of increased drought stress tolerance (Fig. 5E).

The sulfide content was significantly increased in all tomato plants under drought stress compared with the control plants (Fig. 5F). As expected, the sulfide levels were 3-fold higher in the *SiR* OE (which reduces sulfite to sulfide) and *SiR* OE/*SO* OE plants than in the RR and *SO* OE plants. Cysteine, the downstream product of sulfide in the sulfate reduction pathway, was also significantly increased in the *SiR* OE and *SiR* OE/*SO* OE plants compared with the RR and *SO* OE plants under both control and drought stress conditions (Fig. 5G). Notably, H_2_O_2_, which was similar in all tomato plants under control conditions, was significantly enhanced in response to drought stress, with the most pronounced elevation in the RR plants, compared with the three mutant plants, indicating enhanced drought stress response in the RR plants (Fig. 5H). Overall, these results suggest that as in the Arabidopsis mutant plants, the decrease in the sulfite levels through oxidation resulted in an increased sulfate level and sulfate/sulfite ratio in the *SO* OE and *SiR* OE/*SO* OE tomatoes. Additionally, the enhanced sulfite reduction through *SiR* OE, which decreased sulfite and increased sulfide levels in the *SiR* OE and *SiR* OE/*SO* OE tomatoes, further improved drought tolerance in these mutants compared with the WT (RR) plants.

### Similar to Arabidopsis, the response of tomato plants to drought is associated with the interaction of abscisic acid with other metabolites

ABA is an essential phytohormone for controlling stomatal aperture in response to drought stress. Enhancement of ABA biosynthesis in response to drought is initiated by inducing AtNCED3, the committed step of ABA biosynthesis, and finalized in Arabidopsis by the oxidation of the synthesized abscisic aldehyde to ABA by AAO3. AAO3 is activated by the sulfuration of its molybdenum cofactor active center by ABA deficient 3 (ABA3) using L-cysteine, the product of the sulfate reduction pathway (Sagi *et al*., 1999, 2002; Ernst *et al*., 2010; Malcheska *et al*., 2017; Batool *et al*., 2018). While under control conditions, the ABA levels were similar in all plants present in each experiment (Figs 2H, 3I, 6A), in response to drought stress, ABA significantly increased in Arabidopsis *SO* Ri compared with the WT. In addition, both the *SO* Ri and WT plants exhibited higher ABA enhancement than the *SO* OE, *apr2* KO, and *apr2* KO/*SO* OE plants (Fig. 2H). Similarly, while exhibiting similar ABA levels under unstressed control conditions, in response to drought stress, the WT exhibited a higher ABA increase than the increase in the various Arabidopsis double mutants overexpressing *SiR* and *SO* (Fig. 3I). Similarly, while the RR, *SO* OE, *SiR* OE, and *SiR* OE/*SO* OE tomato plants showed similar ABA levels under control conditions, all tomato genotypes showed a significant increase in ABA under drought stress, with RR plants showing substantially higher ABA levels than the other genotypes (Fig. 6A). Notably, similar to Arabidopsis, in which the WT and *SO* Ri exhibited higher ABA levels but lower RWC than the other examined genotypes (Figs 2H, 3I), the lower ABA enhancement in the tomato mutants was enough to lead to a higher RWC than the higher ABA level in the RR (WT) under drought conditions. As previously shown, sulfite accumulation limited stomatal closure, decreasing RWC in Arabidopsis leaves (Bekturova *et al*., 2021). Similarly, the highest sulfite levels in the RR plant leaves resulted in lower RWC, even with higher ABA levels, than in the *SO* OE, *SiR* OE, and *SiR* OE/*SO* OE mutant leaves (Figs 5A, B, 6A).

**Fig. 6.**
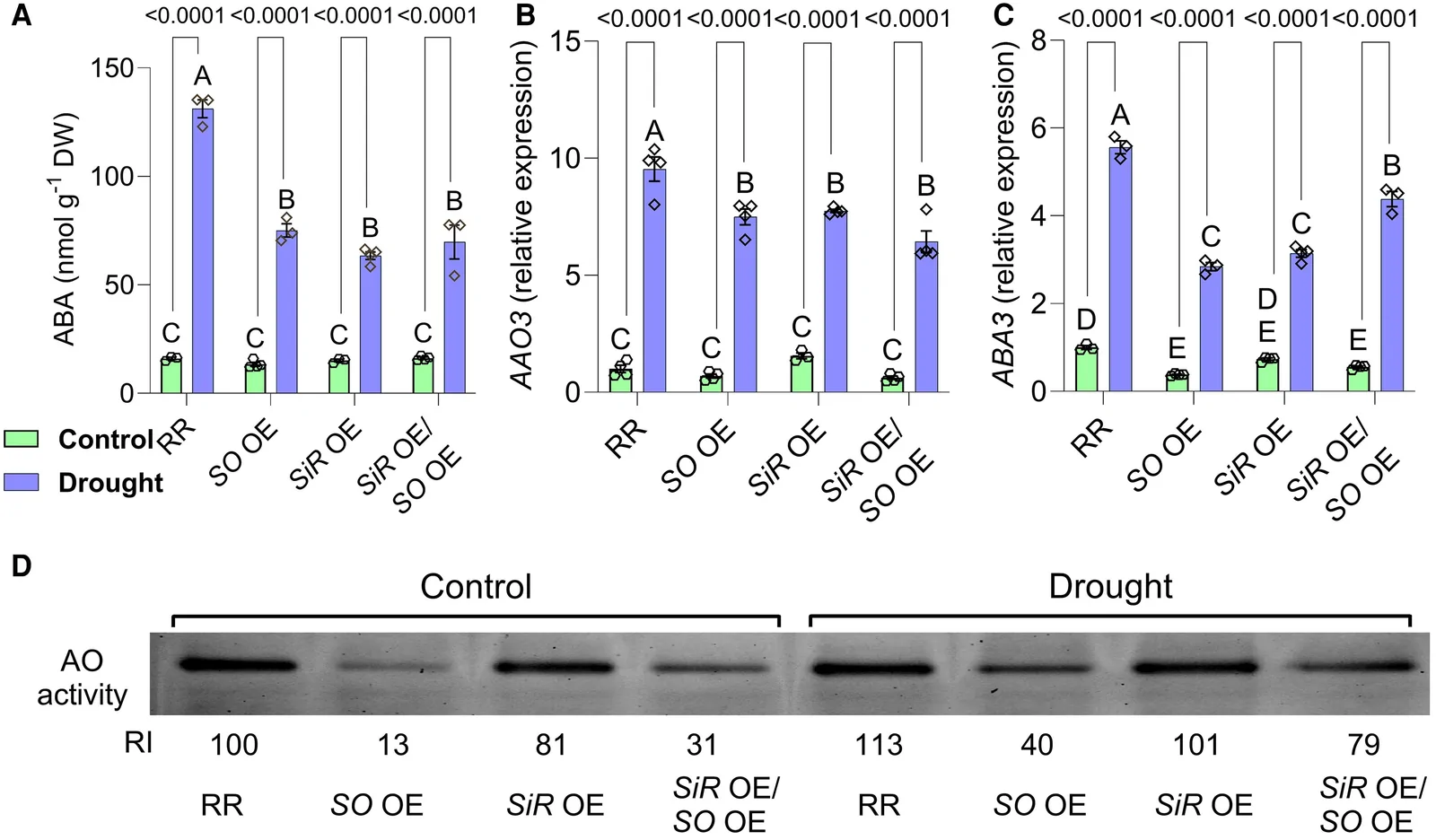
Effects of drought stress on ABA content, ABA-related genes, and aldehyde oxidase (AO) activity of tomato plant in Rheinlands Ruhm (RR; wild type), *sulfite oxidase* overexpression (*SO* OE), *sulfite reductase* overexpression (*SiR* OE), *SiR* OE, and *SO* OE double overexpression (*SiR* OE/*SO* OE) mutant plants. (A) ABA content [nmol g^−1^ dry weight (DW)]. (B) *Aldehyde oxidase 3* (*AAO3*) gene expression. (C) *Abscisic acid deficient 3* (*ABA3*) gene expression. (D) AO activity. Two-way ANOVA followed by Tukey’s multiple comparison test was applied to all panels. Significant main effects of genotype and treatment (both *P*<0.0001) were detected across all panels. Genotype×treatment interaction *P*-values were as follows: ABA (*P*<0.0001), *AAO3* gene expression (*P*=0.0006), *ABA3* gene expression (*P*<0.0001). Different uppercase letters indicate significant differences among genotypes regardless of treatment (*n*=4).

*SO* and *SiR* overexpression also affected gene expression related to ABA biosynthesis. Compared to the RR plants under drought stress, in keeping with the lower ABA level, the relative expression of *AAO3* and *ABA3* significantly decreased in the *SO* OE, *SiR* OE, and *SiR* OE/*SO* OE tomato plants (Fig. 6B, C). Further, in-gel activity, using abscisic aldehyde as the specific substrate for AAO3 in Arabidopsis (Srivastava *et al*., 2017; Nurbekova *et al*., 2021, 2024 and references therein), confirmed that aldehyde oxidase 3 (AO3) activity was lower in the *SO* OE, *SiR* OE, and *SiR* OE/*SO* OE tomato plants than in the RR plants under both control and drought stress conditions (Fig. 6D). The enhanced AO3 activity in the RR leaves is supported by the transcript expression of *AO3* and *ABA3*, the latter of which is responsible for activating aldehyde oxidases by sulfuration of the molybdenum cofactor site in the AO3 enzyme (Sagi *et al*., 1999, 2002). Importantly, further support of the validity of the results related to ABA is that similar to ABA, the inactive derivatives of ABA—phaseic acid and neophaseic acid—decreased significantly in the *SO* OE, *SiR* OE, and *SiR* OE/*SO* OE tomato plant leaves, compared with the RR plant leaves, under both control and drought stress conditions (Supplementary Fig. S6A, B). Similarly, (+)-7-OH-ABA and ABA glucose ester levels were significantly lower in the *SO* OE, *SiR* OE, and *SiR* OE/*SO* OE tomato leaves than in the RR leaves under drought stress (Supplementary Fig. S6C, D).

## Discussion

### The major drought stress indicators are sulfite and sulfate rather than sulfide or L-cysteine

Drought is a key environmental stress affecting crop productivity worldwide (Zhang *et al*., 2025). Plant sulfur metabolism components, such as sulfate, L-cysteine, and glutathione, have been thoroughly investigated for crop improvement under drought stress (Chan *et al*., 2013; Capaldi *et al*., 2015; Takahashi *et al*., 2023). The source of sulfide, which is considered to play a role in drought tolerance by controlling stomatal aperture and senescence-triggered autophagy (Álvarez *et al*., 2012; Jin *et al*., 2013; Scuffi *et al*., 2014; Malcheska *et al*., 2017; Wang *et al*., 2024), originates not only from SiR activity but also from L-cysteine degradation by L-cysteine desulfhydrase 1 (Álvarez *et al*., 2010; Romero *et al*., 2014), as well as from L-cysteine degradation by the three OASTLs in Arabidopsis (Kurmanbayeva *et al*., 2022; Sun *et al*., 2025). Yet, the effects of sulfite homeostasis on plant resistance to drought stress remain comparatively underexplored. The current study examined mutants of genes involved in sulfite homeostasis in Arabidopsis and tomato to examine drought stress responses.

Importantly, the current study employed a significant number of reliable mutant plants. Among three independent *SiR* OE/*SO* OE lines, two used the CRISPR–Act 3.0 system; all three showed results consistent with expectations. At the same time, the single mutants of Arabidopsis *SO* Ri and *SO* OE, as well as the tomato *SO* OE and *SiR* OE, were examined before with at least one additional independent line not demonstrating any positional effects and somaclonal variation (Brychkova *et al*, 2007; Yarmolinsky *et al*., 2013). Additionally, the tomato *SiR* OE/*SO* OE showed results similar to those of the Arabidopsis double mutants under drought stress. As for the *apr2* KO single and *apr2* KO/*SO* OE, both showed the absence of APR protein expression, lower H_2_O_2_ and ABA levels, and higher RWC compared with WT, supporting the reliability of the mutants used (Figs 1–6). Taken together, the convergence of results across multiple independent genetic manipulations and two plant species provides strong confidence in the robustness of the present findings.

The contribution of the interplay between SO, SiR, and APR2 expression levels and sulfite-induced water loss levels was demonstrated in Arabidopsis (Bekturova *et al*., 2021; Figs 1–3) and tomato leaves (Figs 4–6). Despite the distinct sulfur metabolism in Arabidopsis and tomato plants (Yarmolinsky *et al*., 2013), both species exhibit analogous physiological responses to drought stress and sulfite toxicity. The higher relative water content, reduced wilting, and lower sulfite levels in the *SO* OE, *apr2* KO/*SO* OE, *SlSiR* OE/*SO* OE, CRISPR (*SiR* OE*^C2^*+*SO* OE*^C2^* and *SiR* OE*^C4^*/*SO* OE) (overexpressing *SiR* and *SO*) plants than in the *SO* Ri and WT plants of Arabidopsis, and in the *SO* OE, *SiR* OE and *SiR* OE/*SO* OE plants than in the RR (WT) plants of tomato suggest a negative correlation between water content and the sulfite level in plant leaves under drought stress. A previous study demonstrated the role of sulfite levels in drought-induced rosette leaf detachment, after preincubation in buffers containing or lacking sulfite or sulfate, and in drought-induced root detachment in Arabidopsis plants. Additionally, it was shown that a 48 h irrigation cessation in 23-day-old Arabidopsis plants, grown in a limited volume of field capacity watered low-nutrient soil, resulted in lower RWC in *SO* Ri than in WT and *SO* OE plants (Bekturova *et al*., 2021). In the current study, it was shown that Arabidopsis mutant plants with *SO* overexpression [SO OE, *apr2* KO/*SO* OE, *SlSiR* OE/*SO* OE, and CRISPR (*SiR* OE*^C2^*+*SO* OE*^C2^* and *SiR* OE*^C4^*/*SO* OE)], as well as tomato mutant plants (*SO* OE and *SiR* OE/*SO* OE), efficiently catalyse the oxidation of sulfite to sulfate. It was also shown that the tomato *SiR OE* effectively catalysed the reduction of sulfite to sulfide (Figs 2A, C, D, 3C–E, 5B, D, E). These results suggested that, in addition to SO and SiR, previously shown to act as a safety valve that protects cells against toxic sulfite levels (Brychkova *et al*., 2007, 2013; Lang *et al*., 2007; Randewig *et al*., 2012; Yarmolinsky *et al*., 2013, 2014), any activity that limits the sulfite accumulation in leaves, such as *apr2* KO, may improve plant response to drought stress. Indeed, in a previous study, *apr2* KO plants exhibited higher RWC and lower stomatal aperture than WT plants when 8 mM sulfite was infiltrated into the leaves (Bekturova *et al*., 2021). However, while *apr2* KO had a lower sulfite level than *SO* Ri, explaining its higher RWC, its enhanced sulfite level, compared with the WT, needs to be attributed to an additional factor, such as its higher sulfate level, to explain the higher RWC in *apr2* KO than in WT under drought conditions (Figs 1B, 2A, C). Indeed, compared with the WT and *SO* Ri in Arabidopsis and RR in tomato, the sulfate enhancement in the mutants most likely acts as an inducer of stomatal closure to prevent water loss in response to drought. In support of this idea, the increase in sulfate level was shown previously to trigger stomatal closure via activation of ABA biosynthesis (Ernst *et al*., 2010; Batool *et al*., 2018). Similarly, higher levels of sulfate in Arabidopsis and maize (Batool *et al*., 2018), common hop (*Humulus lupulus*) (Korovetska *et al*., 2014), and *Populus×canescens* (Malcheska *et al*., 2017) have been shown to play a role in stomatal closure in response to drought stress. In our previous study, sulfate enhancement resulted in a more reduced stomatal aperture and water loss in WT and *SO* Ri, while the decrease was less with enhanced sulfite levels (Bekturova *et al*., 2021). The higher sulfate level in the *apr2* KO mutant than in the WT and *SO* Ri plants (Fig. 2C) is due to the enhanced SO activity in response to drought (Fig. 2B), as well as the impairment in APR2, which acts as a bottleneck, decreasing sulfate reduction to sulfite and thus decreasing sulfite's toxic effects. The imbalance of the sulfur metabolism due to non-functional SO in *SO* Ri, as well as the absence of excess sulfite detoxifiers, such as *SO* OE and *SiR* OE, or the lack of sulfite enhancement preventors, such as APR2 impairment, led to the lowest sulfate levels in *SO* Ri and WT, accompanied by an increase in sulfite. This most likely results in impaired stomatal closure and lower RWC under drought conditions in these plants than in other genotypes.

Sulfide has been shown to support L-cysteine biosynthesis, and elevated L-cysteine aids in ABA biosynthesis (Bittner *et al*., 2001; Cao *et al*., 2014). The source of the generated sulfide levels detected in the leaves of plants exposed to drought stress may be synthesis through SiR activity, using sulfite as the substrate (Randewig *et al*., 2012; Yarmolinsky *et al*., 2013), as well as L-cysteine degradation by L-cysteine desulfhydrase 1 (Álvarez *et al*., 2010; Romero *et al*., 2014), and by the three OASTLs in Arabidopsis (Kurmanbayeva *et al*., 2022). Hydrogen sulfide was shown to play an important role in ABA-mediated stomatal movement in Arabidopsis and crop plants (Ma *et al*., 2016; Jin *et al*., 2017; Chen *et al*., 2020; Zhou *et al*., 2020; Jurado-Flores *et al*., 2023). In the current study, the lower sulfide level detected in *SO* Ri plants may support the notion that the relatively low sulfide level, together with the low sulfate and high sulfite levels, likely affected stomatal opening, resulting in lower RWC in leaves of *SO* Ri plants under drought stress. In contrast, Arabidopsis WT and *apr*2 KO plants exhibited similar sulfide levels, while *apr2* KO had a significantly higher RWC than WT in response to drought stress (Fig. 2). Similarly, RR, the tomato WT, exhibited a similar sulfide level as the tomato *SO* OE. In comparison, the mutant leaves had significantly higher RWC and higher sulfate and lower sulfite levels, indicating that sulfite and sulfate levels in leaves play a significant role in protecting RWC in plants exposed to drought stress (Fig. 5).

The accumulation of L-cysteine in response to sulfite stress might be caused by the reduction of sulfite to sulfide by SiR and the subsequent L-cysteine biosynthesis or the conversion of sulfite-induced degraded protein, resulting in free L-cysteine (Ranieri *et al*., 1995; Wen *et al*., 1996). Like sulfide, the possibility of L-cysteine acting as an indicator for drought stress in plants was examined. In contrast to *SO* Ri having the lowest sulfide level, compared with the other plants under drought stress, the highest L-cysteine level was detected in the *SO* Ri plant leaves under drought stress (Fig. 2E, F), while these plants exhibited the lowest SiR expression level (Fig. 2B). These results indicate that the L-cysteine level is most likely associated with protein degradation because of the deleterious effects of the high sulfite content (Fig. 2A), resulting in the lowest RWC in response to drought stress, while exhibiting the highest L-cysteine level (Figs 1B, 2F). In contrast, *apr2* KO, which, similarly to *SO* Ri, had a decrease in sulfide level (Fig. 2E) but a higher RWC than *SO* Ri and WT under drought stress (Fig. 1B), exhibited an enhanced L-cysteine level, as in the WT leaves in response to the stress (Fig. 2F). This further indicates that the major drought stress indicators are the sulfite and sulfate levels, rather than sulfide or L-cysteine.

### Enhanced sulfite in the presence of low sulfate acts as an antagonist to abscisic acid and hydrogen peroxide

Our previous study demonstrated that sulfite infiltration into Arabidopsis leaves activates NADPH oxidase, thereby enhancing H_2_O_2_ production and increasing APR activity, which in turn generates more sulfite. In contrast, sulfite levels decreased due to increased sulfite oxidation by *SO* OE, suppression of APR2 by a T-DNA mutation, and inhibition of the FAD-containing NADPH oxidase with diphenyleneiodonium, which reduced sulfite production by APR. This sulfite decrease caused a reduction in stomatal aperture and a higher RWC than in the sulfite-infiltrated *SO* Ri mutant and WT (Bekturova *et al*., 2021). Similarly, the higher sulfite enhancement induced by the applied long-term drought stress in Arabidopsis *SO* Ri and WT and the tomato RR was also followed by a higher H_2_O_2_ increase than in the other mutant plants (*SO* OE, *apr2* KO, *apr2* KO/*SO* OE, *SlSiR* OE/*SO* OE, CRISPR (*SiR* OE*^C2^*+*SO* OE*^C2^* and *SiR* OE*^C4^*/*SO* OE), as well as the tomato mutant plants *SO* OE, *SiR* OE and *SiR* OE/*SO* OE, all of which showed better resistance to the drought conditions. The enhanced drought tolerance, reflected by the higher RWC (Figs 2, 3, 5), was most likely due to the lower sulfite levels in leaves, which have been shown to induce an increase in stomatal aperture, resulting in higher water loss and lower RWC (Bekturova *et al*., 2021). The lower H_2_O_2_ in *apr2* KO, although the plants exhibited higher sulfite levels than WT under drought stress, can be explained by the protection conferred by the plant’s higher sulfate level, as discussed above (Fig. 2A, C, G). In the current study, the *SO* OE, *apr2* KO, *apr2* KO/*SO* OE, *SlSiR* OE/*SO* OE, *SiR* OE*^C2^*+*SO* OE*^C2^*, and *SiR* OE*^C4^*/*SO* OE mutants in Arabidopsis, and *SO* OE, *SiR* OE, and *SiR* OE/*SO* OE in tomato showed significantly higher sulfate levels and lower sulfite levels than WT and *SO* Ri plants under drought stress, resulting in higher RWC and reduced wilting symptoms. These results indicate that the RWC depends on the sum of the two opposite effects, that is, the protective effect of sulfate and the damaging effect of sulfite accumulation, as demonstrated before in Arabidopsis (Bekturova *et al*., 2021) and in the current study in Arabidopsis and tomato plants exposed to drought stress (Figs 1B, 2A, C, 3B–D, 5A, B, D).

The gene expression data (Supplementary Fig. S2) provide secondary molecular evidence consistent with most likely impaired stomatal regulation in *SO* Ri. Since WT also loses RWC without substantially inducing these genes, the primary determinants of differential water retention remain the sulfite accumulation and the sulfate/sulfite ratio (Figs 1, 2). Relative to WT under drought, *SO* Ri shows specific suppression of *PYL5* to the absolute lowest value of all genotypes (Supplementary Fig. S2A), the strongest *HAI2* induction, reflecting the highest ABA-driven negative feedback (Supplementary Fig. S2B; Ma *et al*., 2009; Munemasa *et al*., 2015), and the most severe suppression of *KAT1* of all genotypes (Supplementary Fig. S2F; Kim *et al*., 2010; Hua *et al*., 2012), together indicating impaired ABA perception and guard cell ion transport most likely contributing to the highest water loss and lowest RWC in *SO* Ri (Figs 1B, 2A). The convergence of drought-induced sulfite accumulation, RWC differences, evidence of ABA antagonism, and the directly established sulfite–stomatal relationship (Bekturova *et al*., 2021) provides strong mechanistic support for the conclusion that sulfite-mediated impairment leads to differential water loss, most likely due to failure of stomatal closure across these genotypes under prolonged drought stress.

In our previous study, while applying ABA to WT rosette leaves decreased stomatal aperture, further application of sulfite to the ABA-treated leaves enhanced stomatal aperture, demonstrating that the sulfite acts as an antagonist to ABA-induced stomatal closure (Bekturova *et al*., 2021). In the current study, this antagonism was confirmed in intact plants exposed to prolonged drought stress. It was clearly shown in the Arabidopsis and tomato plants that the higher sulfite generation in response to drought stress resulted in enhanced ABA production in Arabidopsis *SO* Ri (Fig. 2A, H) and WT **(**Figs 2A, H, 3C, I), as well as in the tomato RR (Figs 5B, 6A), as compared with the other mutant plants, whose targeted mutations successfully decreased sulfite accumulation while causing higher RWC than in the Arabidopsis *SO* Ri and WT and tomato RR (WT) plants (Figs 1B, 3B, 5A). Notably, despite exhibiting elevated ABA levels, the *SO* Ri and RR plants showed the lowest RWC under drought stress, clearly demonstrating that sulfite accumulation antagonizes ABA-mediated responses and impairs water retention. The similar sulfite levels in the WT and *apr2* KO leaves, while these plants have higher RWC and lower ABA levels, are the result of the higher sulfate level that counteracts the effect of sulfite, further supporting the notion that the sulfate/sulfite ratio, rather than ABA level alone, is a key determinant of RWC under drought stress (Figs 1B, 2A, C, H, 3B, C, I).

## Supplementary Material

erag344_Supplementary_Data

## Data Availability

All data supporting the findings of this study are available within the paper and within its supplementary materials published online. Raw uncropped western blot and gel images are provided in Supplementary Fig. S7. Sequence data for Arabidopsis used in this work can be found in the GenBank/EMBL data libraries under accession numbers AT5G60390 [elongation factor (EF)1-α]. Sequence data for tomato used in this work can be found in the SGN data libraries under accession numbers SGN-U196120 [Ef1-α].

## Supplementary data

The following supplementary data are available at *JXB* online.

Fig. S1. Effects of drought stress on total protein and ABA derivatives in wild type (WT), *sulfite oxidase* overexpression (*SO* OE), *SO* RNA interference (*SO* Ri), *5′-adenylylphosphosulfate reductase 2* knockout (*apr2* KO), and *apr 2* KO/*SO* OE Arabidopsis leaves.

Fig. S2. Effect of drought stress on the relative expression of ABA downstream signaling and stomatal closure-related genes in Arabidopsis.

Fig. S3. Effects of drought stress on the phenotypes of WT, tomato *SiR* overexpression in an Arabidopsis *SO* OE background (*SlSiR* OE/SO OE), Arabidopsis *SiR* and *SO* in a Col-0 background (*SiR* OE*^C2^*+*SO* OE*^C2^*), and Arabidopsis *SiR* in a *SO* OE background (*SiR* OE*^C4^*/*SO* OE) grown under control and drought stress (sixth day).

Fig. S4. Effects of drought stress on relative expression and ABA derivatives in wild type (WT), tomato *SiR* overexpression in an Arabidopsis *SO* OE background (*SlSiR* OE/*SO* OE), Arabidopsis *SiR* and *SO* in a Col-0 background (*SiR* OE*^C2^*+*SO* OE*^C2^*) and Arabidopsis *SiR* in an *SO* OE background (*SiR* OE*^C4^*/*SO* OE) grown under control and drought stress (sixth day).

Fig. S5. Verification of tomato mutants by PCR analysis and western blot analysis.

Fig. S6. Effects of drought stress on ABA derivatives on tomato plants in RR (wild type), *sulfite oxidase* overexpression (*SO* OE) mutants, *sulfite reductase* overexpression (*SiR* OE) mutants, and *SiR* OE and *SO* OE double overexpression (*SiR* OE/*SO* OE) mutants.

Fig. S7. Uncropped western blot and in-gel images.

Table S1. Details of Arabidopsis and tomato mutants used during this study.

Table S2. Primer sequences used for CRISPR construct preparations.

Table S3. Primers used in qRT-PCR and verification of plants.

Table S4. Reference gene stability analysis of elongation factor (EF)1-α using TOST equivalence testing.

Protocol S1. Generation of overexpressed SiR and SO in Arabidopsis by CRISPR technology.

## Abbreviations

7′-OH-ABA: 7′-hydroxy-ABA
ABA: abscisic acid
ABAGE: ABA-glucose ester
APR2/*apr2*: adenosine-5′-phosphosulfate reductase 2
DW: dry weight
KO: knockout
neoPA: neophaseic acid
OASTL: *O*-acetyl serine (thiol)lyase
OE: overexpression
PA: phaseic acid
Ri: RNA interference
RWC: relative water content
SiR: sulfite reductase
SO: sulfite oxidase
