# Supplementary material for: Sulfite dynamics affect drought tolerance and water status in Arabidopsis and tomato
Source: J Exp Bot. 2026 Jul 10;77(19):6756–73. doi: 10.1093/jxb/erag344 (PMC13672997; doi:10.1093/jxb/erag344)
Supplement: Supplementary file 1 [file erag344_supplementary_data.pdf]

Supplementary Information

Supplementary Figures:

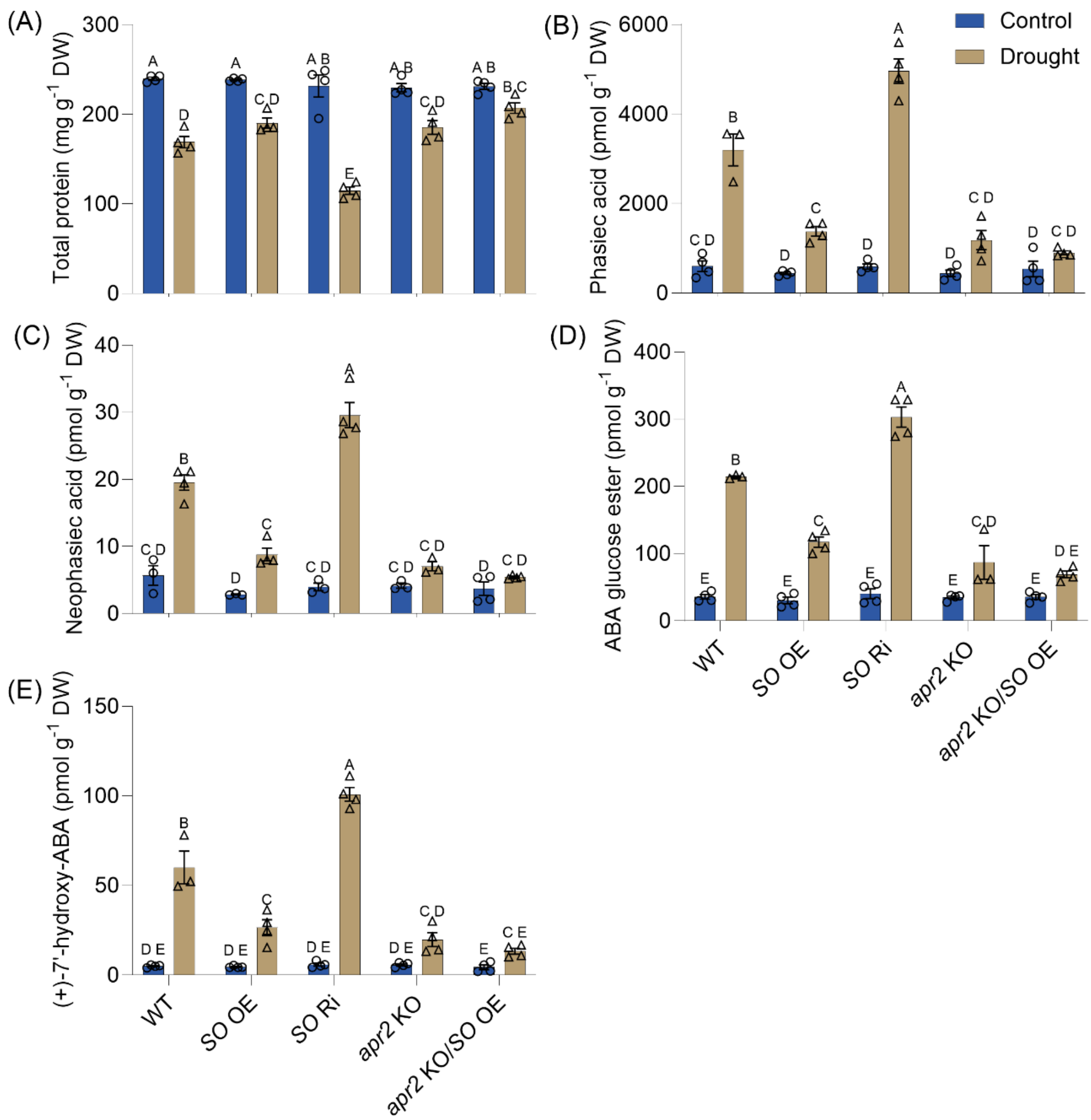

Supplementary Fig. S1: Effects of drought stress on total protein and ABA derivatives in wild type (WT), sulfite oxidase overexpression (*SO* OE), *SO* RNA interference (*SO* Ri), 5'adenylylphosphosulfate reductase 2 knockout *(apr2* KO), and *apr* 2KO/*SO* OE *Arabidopsis* leaves. (A) Total protein (mg g^{-1} DW), (B) Phaseic acid (pmol g^{-1} DW), (C) Neophaseic acid (pmol g^{-1} DW), (D) ABA glucose ester (pmol g^{-1} DW) and (E) (+)-7′-hydroxy-ABA (pmol g^{-1} DW) under control and drought stress (sixth day). Two-way ANOVAs with Tukey's multiple comparison tests were used for identifying the significant difference in values (Different uppercase letters indicate significant differences in genotypes regardless of treatment) (n = 4).

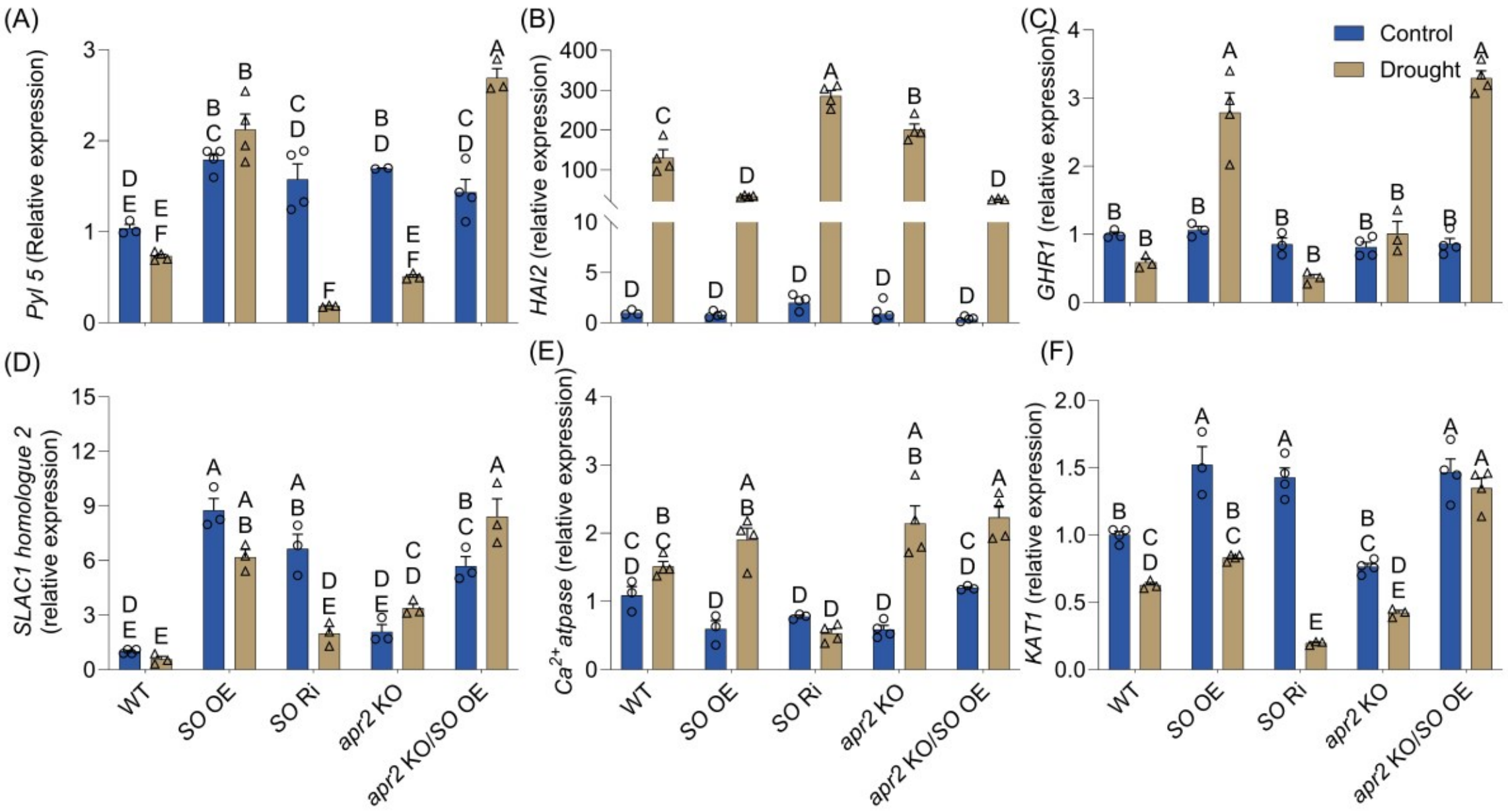

Supplementary Fig. S2: Effect of drought stress on the relative expression of ABA downstream signaling and stomatal closure-related genes in *Arabidopsis* wild type (WT), sulfite oxidase overexpression (*SO* OE), SO RNA interference (*SO* Ri), 5'-adenylylphosphosulfate reductase 2 knockout (*apr2* KO), and *apr2* KO/*SO* OE plants grown under control and drought stress (sixth day). (A) Pyrobactin resistance 1-like 5 (PYL5), (B) Highly ABA-induced PP2C gene 2 (HAI2), (C) Guard cell hydrogen peroxide-resistant 1 (GHR1), (D) SLAC1 homologue 2 (SLAH2), (E) Ca^{2+} ATPase and (F) Hyperpolarization-activated inward-rectifying potassium channel (KAT1). Two-way ANOVAs with Tukey's multiple comparison tests were used for identifying the significant difference in values (Different uppercase letters indicate significant differences in genotypes regardless of treatment) (n = 4).

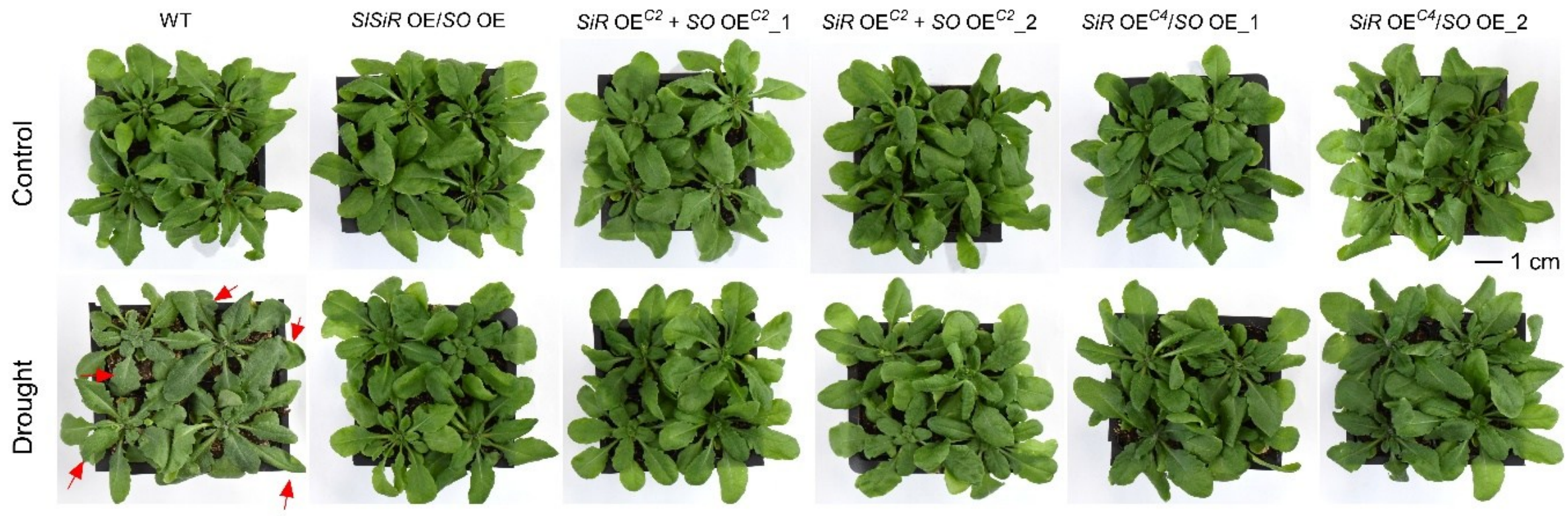

Supplementary Figure S3: Effects of drought stress on the phenotypes of *Arabidopsis* WT, *Solanum lycopersicum* (Sl) sulfite reductase (SiR) overexpression in an *Arabidopsis SO* OE background (*SlSiR* OE/SO OE), *Arabidopsis SiR* and SO overexpression in a Col-0 background (*SiR* OE^{C2} + *SO* OE^{C2}) and *Arabidopsis SiR* overexpression in an *Arabidopsis SO* OE background (*SiR* OE^{C4}/*SO* OE) grown under control and drought stress (sixth day). ^{C2}: C indicates CRISPR, and 2 means two guide RNA for SiR or two guide RNA for SO. ^{C4}: C indicates CRISPR, and 4 indicates four guide RNA for SiR (red arrow indicates wilting).

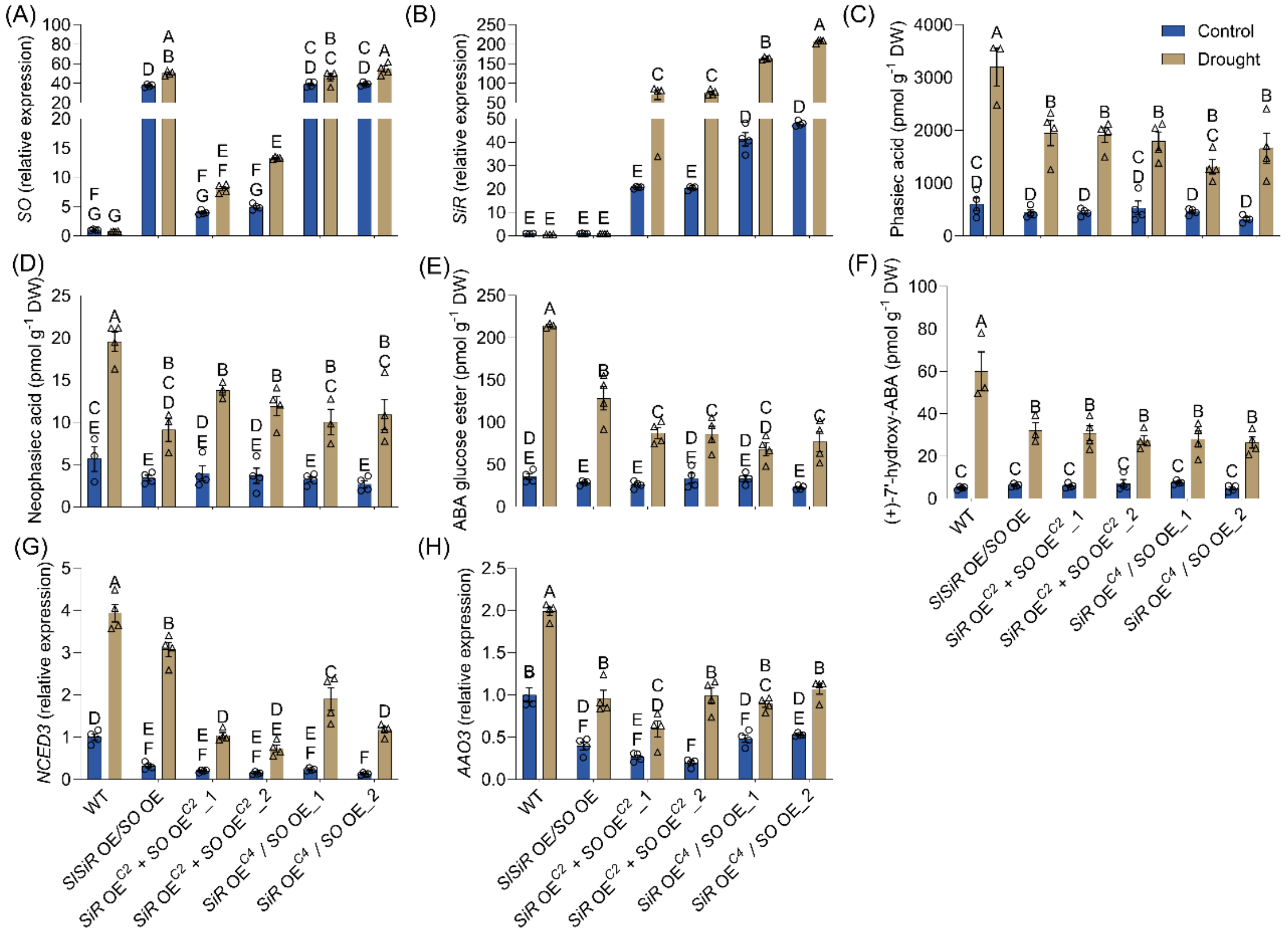

Supplementary Fig. S4: Effects of drought stress on relative expression and ABA derivatives in *Arabidopsis* WT, *Solanum lycopersicum* *SiR* overexpression in an *Arabidopsis SO* OE background (*SlSiR* OE/SO OE), *Arabidopsis SiR* and SO overexpression in a Col-0 background (*SiR* OE^{C2} + *SO* OE^{C2}) and *Arabidopsis SiR* overexpression in an *SO* OE background (*SiR* OE^{C4}/*SO* OE) grown under control and drought stress (sixth day). (A) Sulfite oxidase (*SO*) relative expression, (B) Sulfite reductase (*SiR*) relative expression, (C) Phaseic acid (pmol g^{-1} DW), (D) Neophaseic acid (pmol g^{-1} DW), (E) ABA glucose ester (pmol g^{-1} DW), (F) (+)-7′-hydroxy-ABA (pmol g^{-1} DW), (G) 9-cis-epoxycarotenoid dioxygenase 3 (*NCED3*) relative expression and (H) Aldehyde oxidase (*AAO3*) relative expression. Two-way ANOVAs with Tukey's multiple comparison tests were used for identifying the significant difference in values (Different uppercase letters indicate significant differences in genotypes regardless of treatment) (n = 4).

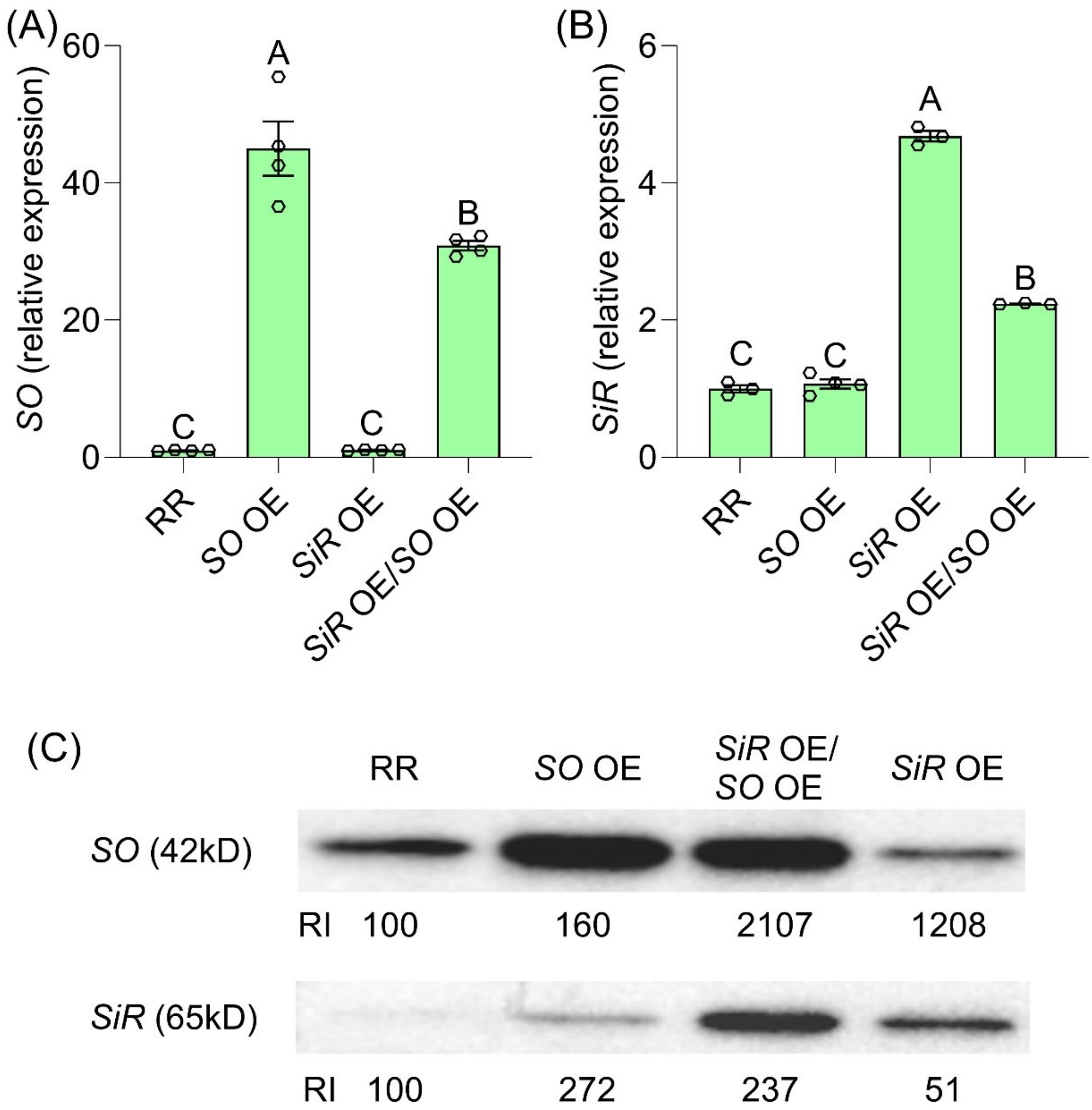

Supplementary Fig. S5: Verification of tomato mutants by PCR and western blot analysis. (A) Sulfite oxidase (*SO*) relative expression (B) Sulfite Reductase (*SiR*) relative expression and (C) Validation of transgenic tomato by western blot using recombinant SiR and SO-specific antibodies (RI = Relative Intensity) in *Solanum lycopersicum* 'Rheinlands Ruhm' (RR) (wild type), sulfite oxidase overexpression (*SO* OE), sulfite reductase overexpression (*SiR* OE), *SiR* OE and *SO* OE double overexpression (*SiR* OE/ *SO* OE) mutant tomato plants. Different uppercase letters indicate significant differences between different genotypes in the same treatment (Tukey-Kramer HSD test and Student's t-test, $P < 0.05$) ($n = 4$).

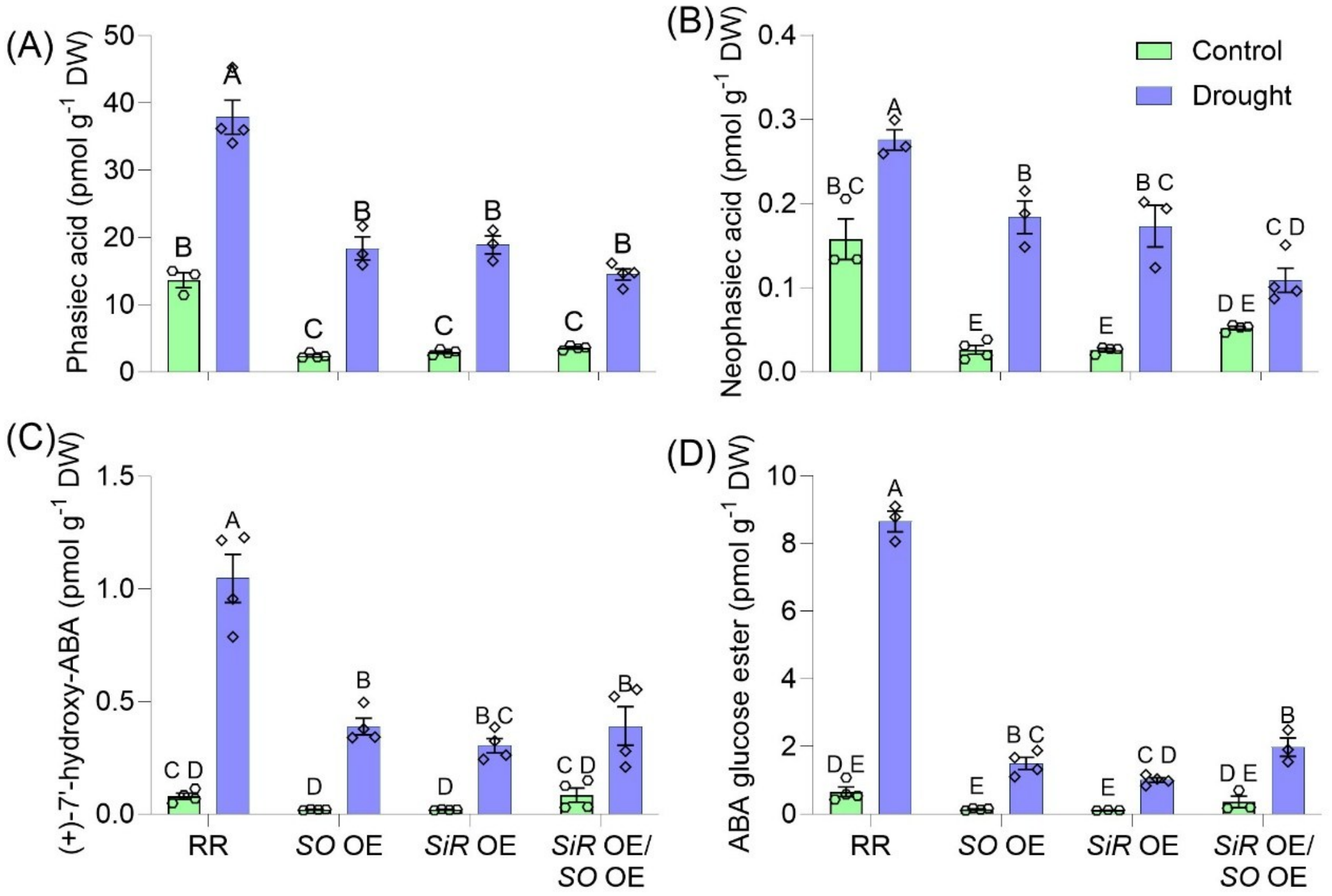

Supplementary Fig. S6: Effects of drought stress on ABA derivatives on tomato plants in RR (wild type), sulfite oxidase overexpression (*SO* OE) mutants, sulfite reductase overexpression (*SiR* OE) mutants, *SiR* OE and *SO* OE double overexpression (*SiR* OE/*SO* OE) mutants. (A) Phaseic acid (pmol g^{-1} DW), (B) Neophaseic acid (pmol g^{-1} DW), (C) (+)-7′-hydroxy-ABA (pmol g^{-1} DW) and (D) ABA glucose ester (pmol g^{-1} DW) of leaves measured in control and drought stress (fourth day). Two-way ANOVAs with Tukey's multiple comparison tests were used for identifying the significant difference in values (Different uppercase letters indicate significant differences in genotypes regardless of treatment) (n = 4).

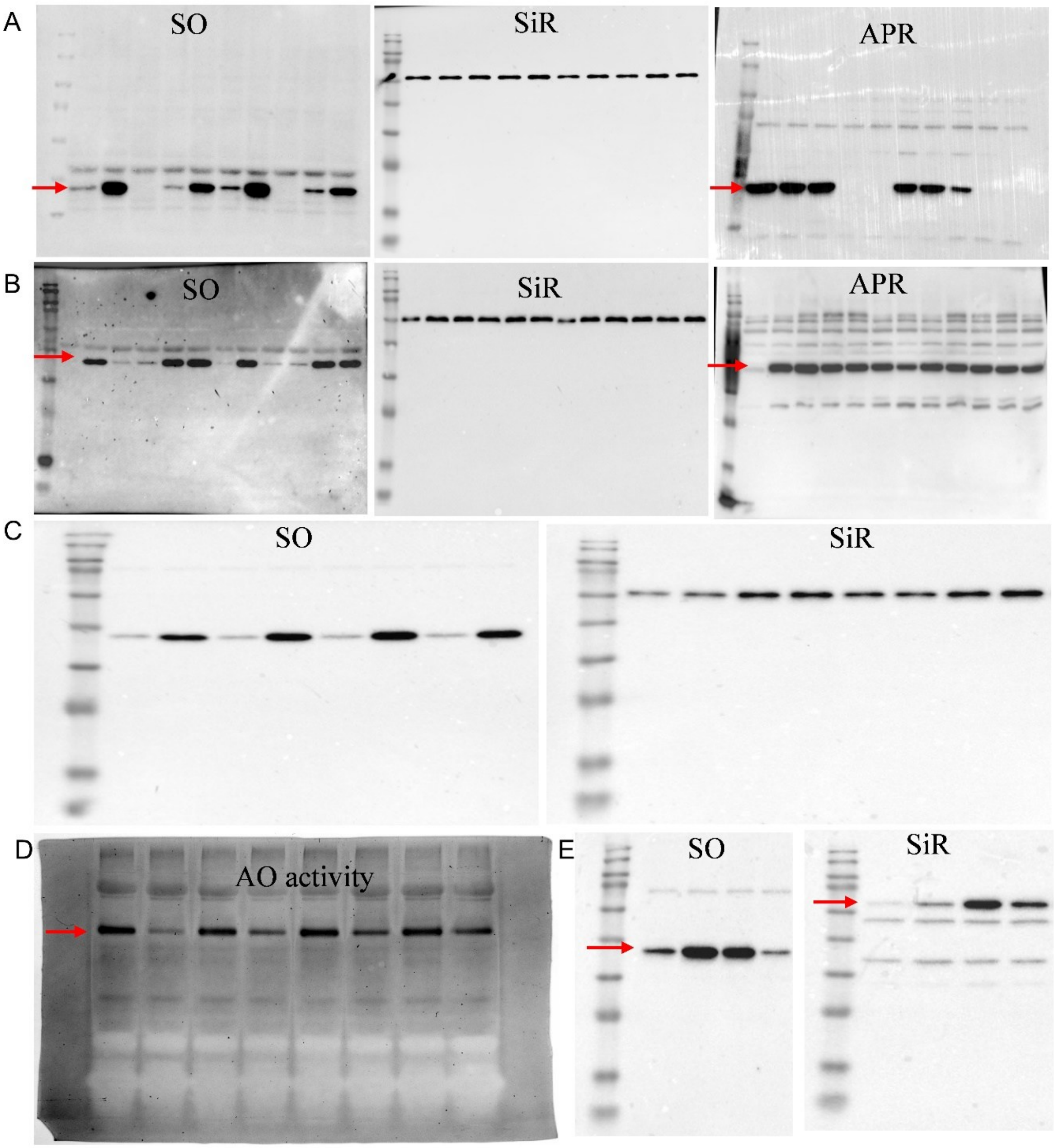

Supplementary Fig. S7: Uncropped western blot and in-gel images. (a) Fig. 2B of (SO, SiR and APR protein), (b) Fig. 3A (SO, SiR and APR protein), (c) Fig. 5C (SO and SiR protein), (d) Fig. 6D (AO activity) and (E) Fig. S4C (SO and SiR protein). Red arrow indicates respective band.

Supplementary Tables:

Supplementary Table S1: Details of *Arabidopsis and* tomato mutants used during this study

Mutant name	Overexpressing or mutant gene	Method of mutation	Genotype background	Reference
Arabidopsis				
SO OE	*Sulfite oxidase* overexpression	Overexpression with CaMV35S promoter	Col-0	Brychkova et al., 2007
SO Ri	*Sulfite oxidase* mutation	Mutation with RNAi	Col-0	Brychkova et al., 2007
apr2 KO	*5'-adenylylsulfate reductase 2* (*apr2*) knockout	T-DNA insertion mutation	Col-0	Loudet et al., 2007
apr2 KO/*SO* OE	*5'-adenylylsulfate reductase 2* (*apr2*) knockout	Cross pollination of *apr2* KO with *SO* OE lines	*SO* OE	NA
SlSiR OE/*SO* OE	*Solanum lycopersicum Sulfite Reductase overexpression*	Overexpression with CaMV35S promoter	*SO* OE	NA
SiR OE^{C2} + *SO* OE^{C2}	*Sulfite Reductase and Sulfite oxidase*	Overexpression with CRISPR, 2*SiR* sgRNA and 2*SO* sgRNA	Col-0	NA
SiR OE^{C4}/*SO* OE	*Sulfite Reductase*	Overexpression with CRISPR, 4SiR sgRNA	*SO* OE	NA
Tomato				
SO OE	*Sulfite oxidase* overexpression	Overexpression with CaMV35S promoter	RR	Brychkova et al., 2007
SiR OE	*Sulfite Reductase* overexpression	Overexpression with CaMV35S promoter	RR	Brychkova et al., 2012b
SiR OE/ *SO* OE	*Sulfite Reductase and Sulfite oxidase* overexpression	Overexpression with CaMV35S promoter	*SO* OE in RR	NA

Here NA indicate mutant developed and used for the first time during this research.

Supplementary Table S2: Primer sequences used for CRISPR construct preparations.

**Construct name**	**gRNA ENTRY VECTOR**	**Insert sequence (F and R primer)**	
Construct 1	pYPQ131B2.0	SIR G1 F	ggtcAGTTGGATTCTCAATGGACGG
		SIR G1R	aaacCCGTCCATTGAGAATCCAACT
	pYPQ132B2.0	SIR G2 F	gattATATCACGTGCATGACACGTG
		SIR G2 R	aaacCACGTGTCATGCACGTGATAT
	pYPQ133B2.0	SO G1 F	ggtcACATACCTCCGCCGTGAAGTT
		SO G1 R	aaacAACTTCACGGCGGAGGTATGT
	pYPQ134B2.0	SO G2 F	ggtcAGCACTCAATCGTCATTTCCT
		SO G2 R	aaacAGGAAATGACGATTGAGTGCT
Construct 1	pYPQ131B2.0	SIR G1 F	ggtcAGTTGGATTCTCAATGGACGG
		SIR G1R	aaacCCGTCCATTGAGAATCCAACT
	pYPQ132B2.0	SIR G2 F	gattATATCACGTGCATGACACGTG
		SIR G2 R	aaacCACGTGTCATGCACGTGATAT
	pYPQ133B2.0	SIR G3 F	ggtcAGGTGGGTGAGAAATCGAATA
		SIR G3 R	aaacTATTCGATTTCTCACCCACCT
	pYPQ134B2.0	SIR G4 F	ggtcATATATCCAAGATGCCATCC
		SIR G4 R	aaacGGATGGCATCTTGGATATAT

Table S3: Primers used in qRT-PCR and verification of plants.

Transcript	Accession Number/ Tair ID	Primer sequence (5'->3')	PCR product bp	PCR condition
Primers for *Arabidopsis thaliana*				
Elongation factor 1-alpha (EF1-α)	AT5G60390	Forward: CAGGACATCGTGATTTCATCAAG	190	1 cycle 95 °C 3 min, 40 cycles 95 °C 15 sec 60 °C 45 sec, Melt curve analysis
		Reverse: TCCATCTTGTTACAACAGCAAAT		
SO (Sulfite oxidase)	AT3G01910	Forward: GTGTGATTGGTGCTCGTTCG	197	
		Reverse: TTGCACATCCTCCACAGAGC		
SiR (Sulfite reductase)	AT5G04590	Forward: GTTGGTGGGGGTATGGGAAG	150	
		Reverse: ACGATCATCTCGTCTCCCGT		

NCED3 (9-cis-epoxycarotenoid dioxygenase NCED3, chloroplastic)	AT3G14440	Forward: TCGAACCGACCAACGGTTTT	160	
		Reverse: GAGCTGCAGCCGGTATAGTCG		
AAO3 (Arabidopsis Aldehyde oxidase 3)	AT2G27150	Forward: GTTGGAGCTGCCTTACAAGCTTCT	151	
		Reverse: CAGTAGGAGTTACATTCTCTCTGAAGC		
PYL5 (Abscisic acid receptor PYL5)	AT5G05440	Forward: TTAGTGTCGTTGGTGGGGAC	180	
		Reverse: AGAGACTGAAGGTTGCACCG		
HAI2 Highly aba-induced PP2C gene 2)	AT1G07430	Forward: CATGGCTGTTCTCACGTTGC	150	
		Reverse: TTCACCCCAACGAACAACCT		
SLAH2 (slac1 homologue 2)	AT4G27970	Forward: CTCGGTTCGCGTCTAGCATA	171	
		Reverse: CACTCCCGTGACTTCGTCTG		
GHR1 (Guard cell hydrogen peroxide-resistant 1)	AT4G20940	Forward: ACGGGGATCTTCCTGGTGTA	166	
		Reverse: AAGTTGCTTGGGAGTGGTCC		
Ca^{2+} ATPase	AT4G00900	Forward: ATGTCTCTTCCGGCTTCACG	190	
		Reverse: TGGCGATACCAATGTCAGCA		
KAT1 (potassium channel protein)	AT5G46240	Forward: TGGGATGGGAAGAGTGGAGA	197	
		Reverse: AGCGTAACTTCTTCCAGCGG		
Primers for tomato (*Solanum lycopersicum*)				
Ef1-α	SGN-U196120	forward CCTACTTGAGGCTCTTGACCAGATT	160	1 cycle 95 °C 3 min, 40 cycles 95 °C 15 sec
		reverse		

		AAAAGTGACAACCATACCA GGCTTAAT		60 °C 45 sec, Melt curve analysis
SO (Sulfite oxidase)	DQ853413	Forward: CCTGGAGGATGTGAGTGTTG TAAAG	145	
		Reverse: AGTTCTCTGGTATCTGGTGG CTTC		
SiR (Sulfite reductase)	SGN-U214723	forward: AAGTTGTGAAAGCTCGGAAT GATAACT	185	
		reverse: TTCTCCATCCTCATCAGATA CAACAAC		
ABA3 (molybdenum cofactor sulfurase (FLACCA)	NM_001247 215	forward: TTGGGGCTGCGTATAGTTTT ATGTG	140	
		Reverse: TGGTCTCAACTCTCGAGAAA GCACT		
AAO3 (Aldehyde oxidase 3)	AF258810	Forward: GGGAGATTCCAAGGAAGTG AAAG	200	
		Reverse: AGCTTGCACCATTTTCCATC A		

Supplementary Table S4: Reference gene stability analysis using TOST equivalence testing. **Section 1.1:** Raw Cq values and descriptive statistics (Mean Cq, SD, CV%) for the reference gene across all *Arabidopsis thaliana* genotypes, control and drought conditions, and four biological replicates per genotype in Experiments 1 and 2 (CV% = SD/Mean × 100). **Section 1.2:** TOST equivalence test confirming invariant reference gene expression between control and drought in *A. thaliana* ($n = 40$ per group, $df = 78$, both experiments independently). **Section 2.1:** Raw Cq values and descriptive statistics for the reference gene across all *Solanum lycopersicum* (Tomato) genotypes, control and drought conditions, and four biological replicates per genotype in Experiments 1 and 2. **Section 2.2:** TOST equivalence test confirming invariant reference gene expression between Control and Drought in Tomato ($n = 16$ per group, $df = 30$, both experiments independently). **Section 3.1:** Inter-run reproducibility: mean Cq per genotype in Experiment 1 vs Experiment 2 for both species and conditions, with $\Delta Cq = |E1 - E2|$ (overall range 0.003–0.434 Cq). **Section 3.2:** Paired TOST for inter-run reproducibility across genotype means (*A. thaliana*: $n = 10$, $df = 9$; Tomato: $n = 4$, $df = 3$). **All analyses:** Equivalence bounds [−0.5, +0.5] Cq correspond to a maximum $2^{0.5} = 1.414$-fold change at 100% PCR efficiency (Bustin et al., 2009; Bustin et al., 2025; Curis et al., 2019). Equivalence was declared when both one-sided test p-values < 0.05 and the 90% CI of the mean Cq difference fell entirely within [−0.5, +0.5] Cq (Lakens, 2017). The 90% CI is used because TOST performs two one-sided tests each at $\alpha = 0.05$. All eight analyses confirmed both $p < 0.05$ and 90% CI within bounds, demonstrating invariant reference gene expression across all conditions, genotypes, species and independent experimental runs, in accordance with MIQE guidelines and JXB policy. Abbreviations: Cq, quantification cycle; SD, standard deviation; CV, coefficient of variation; TOST, Two One-Sided Tests; CI, confidence interval; SE, standard error; df, degrees of freedom; MIQE, Minimum Information for Publication of Quantitative Real-Time PCR Experiments; PCR, polymerase chain reaction; RT-qPCR, reverse transcription quantitative PCR. Collectively, these metrics demonstrate that EF1-α exhibits stable expression across genotypes, treatments, and independent experiments, supporting its suitability as a reference gene for normalisation under the tested conditions, in line with MIQE recommendations (Bustin et al., 2009; 2025). EF1-α is commonly used as a reference gene in RT-qPCR studies in *Arabidopsis* and tomato, where its use in combination with other housekeeping genes has been shown to yield consistent expression patterns (Dekkers et al., 2012; Li et al., 2015; Yarmolinsky et al., 2013; Bekturova et al., 2021; Tapia et al., 2021; Egea et al., 2018; Gong et al., 2010), supporting its selection for normalisation in the present study.

1.1. Raw Cq values: Reference gene stability in *Arabidopsis thaliana* genotypes

**Condition**	**Genotypes**	**Rep 1**	**Rep 2**	**Rep 3**	**Rep 4**	**Mean Cq**	**SD**	**CV%**
**Experiment 1**								
**Control**	WT	19.93	20.06	19.73	19.85	**19.8925**	0.1387	0.697
	SO OE	20.65	20.05	20.14	20.38	**20.3050**	0.2689	1.324
	SO Ri	20.07	20.08	20.24	20.48	**20.2175**	0.1916	0.947
	apr2 KO	20.27	20.22	20.29	20.50	**20.3200**	0.1236	0.608
	apr2 KO/*SO* OE	19.65	20.01	20.09	20.18	**19.9825**	0.2323	1.162
	SlSiR OE/*SO* OE	20.19	20.07	19.92	19.71	**19.9725**	0.2069	1.036
	SiR OE^{C2}+SO OE^{C2}_1	20.16	20.18	20.15	20.04	**20.1325**	0.0629	0.313
	SiR OE^{C2}+*SO* OE^{C2}_2	19.59	19.64	19.52	19.37	**19.5300**	0.1175	0.602
	SiR OE^{C4}/*SO* OE_1	19.79	19.82	19.58	19.78	**19.7425**	0.1097	0.555
	SiR OE^{C4}/*SO* OE_2	19.78	19.90	19.81	20.02	**19.8775**	0.1078	0.542
**Drought**	WT	20.08	20.06	20.22	20.36	**20.1800**	0.1395	0.691
	SO OE	19.79	20.06	20.12	20.28	**20.0625**	0.2040	1.017
	SO Ri	19.62	19.80	19.69	19.51	**19.6550**	0.1218	0.620
	apr2 KO	20.19	20.02	19.96	19.98	**20.0375**	0.1047	0.522
	apr2 KO/*SO* OE	20.02	20.02	20.09	20.43	**20.1400**	0.1961	0.974
	SlSiR OE/*SO* OE	19.99	20.16	20.06	19.98	**20.0475**	0.0830	0.414
	SiR OE^{C2}+SO OE^{C2}_1	20.27	20.15	20.40	20.30	**20.2800**	0.1030	0.508
	SiR OE^{C2}+*SO* OE^{C2}_2	20.03	20.20	20.08	20.45	**20.1900**	0.1874	0.928
	SiR OE^{C4}/*SO* OE_1	20.40	20.04	20.14	20.14	**20.1800**	0.1541	0.763
	SiR OE^{C4}/*SO* OE_2	20.47	20.61	20.41	20.40	**20.4725**	0.0967	0.473
**Experiment 2**								
**Control**	WT	19.61	19.39	19.35	19.41	**19.4400**	0.1160	0.597
	SO OE	19.83	19.73	19.53	19.73	**19.7050**	0.1258	0.639
	SO Ri	20.59	20.52	20.29	20.58	**20.4950**	0.1401	0.684
	apr2 KO	20.18	20.26	20.10	20.24	**20.1950**	0.0719	0.356
	apr2 KO/*SO* OE	19.19	19.40	19.77	19.57	**19.4825**	0.2468	1.267
	SlSiR OE/*SO* OE	20.17	20.02	19.85	19.93	**19.9925**	0.1372	0.686
	SiR OE^{C2}+SO OE^{C2}_1	19.62	19.74	19.81	19.85	**19.7550**	0.1008	0.510

	SiR OE^{C2}+*SO* OE^{C2}_2	19.63	19.59	19.51	19.55	**19.5700**	0.0516	0.264
	SiR OE^{C4}/*SO* OE_1	19.79	19.75	19.65	19.74	**19.7325**	0.0591	0.299
	SiR OE^{C4}/*SO* OE_2	19.96	19.90	20.04	19.94	**19.9600**	0.0589	0.295
**Drought**	WT	19.77	19.74	19.81	19.76	**19.7700**	0.0294	0.149
	SO OE	20.08	19.97	20.04	20.10	**20.0475**	0.0574	0.286
	SO Ri	19.50	19.47	19.37	19.41	**19.4375**	0.0585	0.301
	apr2 KO	20.29	20.16	20.18	20.05	**20.1700**	0.0983	0.487
	apr2 KO/*SO* OE	20.23	20.19	20.04	20.05	**20.1275**	0.0967	0.481
	SlSiR OE/*SO* OE	20.17	20.16	20.09	20.28	**20.1750**	0.0785	0.389
	SiR OE^{C2}+SO OE^{C2}_1	20.42	20.09	20.43	20.27	**20.3025**	0.1595	0.785
	SiR OE^{C2}+*SO* OE^{C2}_2	20.25	20.38	19.99	20.30	**20.2300**	0.1687	0.834
	SiR OE^{C4}/*SO* OE_1	20.46	20.05	19.98	20.11	**20.1500**	0.2134	1.059
	SiR OE^{C4}/*SO* OE_2	20.55	20.63	20.59	20.26	**20.5075**	0.1682	0.820

1.2.TOST equivalence test for *A. thaliana* genotypes

	**Parameter**	**Experiment 1**	**Experiment 2**
**Sample**	n per group (Control / Drought)	40 / 40	40 / 40
**Descriptive Statistics**	Mean Control Cq	19.9973	19.8328
	Mean Drought Cq	20.1245	20.0918
	SD Control	0.2840	0.3367
	SD Drought	0.2380	0.3052
	**Mean Difference (Control - Drought)**	**-0.1273 Cq**	**-0.2590 Cq**
	Standard Error (SE)	0.0586	0.0719
	Degrees of Freedom (df)	78	78
**Equivalence Parameters**	Equivalence Bound (+/- Δ)	+/- 0.5 Cq	+/- 0.5 Cq
	Fold-change Threshold	2^0.5 = 1.414	2^0.5 = 1.414
**TOST Statistics**	Test 1 t-statistic (diff > -0.5 Cq)	6.3631	3.3544
	**Test 1 p-value**	**6.24 x 10^{-9}**	**6.15 x 10^{-4}**
	Test 2 t-statistic (diff < +0.5 Cq)	10.7075	10.5643
	**Test 2 p-value**	**2.81 x 10^{-17}**	**5.25 x 10^-17**
**Confidence Interval**	t-critical (df=78, 95th percentile)	1.6646	1.6646

	**90% CI of Mean Difference**	**[-0.2248, -0.0297]**	**[-0.3786, -0.1394]**

2.1. Raw Cq values: Reference gene stability in *Solanum lycopersicum* (tomato) genotypes

**Condition**	**Genotypes**	**Rep 1**	**Rep 2**	**Rep 3**	**Rep 4**	**Mean Cq**	**SD**	**CV%**
				**Experiment 1**				
**Control**	RR (WT)	20.36	20.67	20.26	20.36	**20.4125**	0.1780	0.8721
	SO OE	20.23	20.25	20.28	20.64	**20.3500**	0.1944	0.9554
	SiR OE	19.82	19.98	20.09	20.36	**20.0625**	0.2272	1.1325
	SiR OE/*SO* OE	20.13	20.22	20.23	20.43	**20.2525**	0.1266	0.6251
**Drought**	RR (WT)	20.39	20.07	20.34	20.54	**20.3350**	0.1960	0.9641
	SO OE	20.87	20.16	20.43	20.62	**20.5200**	0.3001	1.4625
	SiR OE	20.61	20.21	20.59	20.55	**20.4900**	0.1883	0.9191
	SiR OE/*SO* OE	20.52	20.34	20.49	20.53	**20.4700**	0.0883	0.4314
				**Experiment 2**				
**Control**	RR (WT)	20.68	20.60	20.14	20.49	**20.4775**	0.2381	1.1627
	SO OE	20.42	20.21	20.40	20.40	**20.3575**	0.0988	0.4852
	SiR OE	20.16	20.16	20.51	20.32	**20.2875**	0.1664	0.8202
	SiR OE/*SO* OE	20.25	20.16	20.56	20.48	**20.3625**	0.1884	0.9252
**Drought**	RR (WT)	20.42	20.19	20.35	20.52	**20.3700**	0.1388	0.6814
	SO OE	20.63	20.18	20.57	20.65	**20.5075**	0.2210	1.0775
	SiR OE	20.46	20.06	20.40	20.61	**20.3825**	0.2324	1.1404
	SiR OE/*SO* OE	20.72	20.32	20.73	20.61	**20.5950**	0.1912	0.9285

2.2. TOST Equivalence Test in tomato genotypes

	**Parameter**	**Experiment 1**	**Experiment 2**
**Sample**	n per group (Control / Drought)	16 / 16	16 / 16
**Descriptive Statistics**	Mean Control Cq	20.2694	20.3712
	Mean Drought Cq	20.4537	20.4637
	SD Control	0.2147	0.1757
	SD Drought	0.1993	0.2023
	**Mean Difference (Control - Drought)**	**-0.1844 Cq**	**-0.0925 Cq**
	Standard Error (SE)	0.0732	0.0670
	Degrees of Freedom (df)	30	30

**Equivalence Parameters**	Equivalence Bound (+/- Δ)	+/- 0.5 Cq	+/- 0.5 Cq
	Fold-change Threshold	2^0.5 = 1.414	2^0.5 = 1.414
**TOST Statistics**	Test 1 t-statistic (diff > -0.5 Cq)	4.3097	6.0821
	**Test 1 p-value**	**8.10 x 10^-5**	**5.54 x 10^-7**
	Test 2 t-statistic (diff < +0.5 Cq)	9.3447	8.8433
	**Test 2 p-value**	**1.08 x 10^-10**	**3.69 x 10^-10**
**Confidence Interval**	t-critical (df=78, 95th percentile)	1.6973	1.6973
	**90% CI of Mean Difference**	**[-0.3087, -0.0601]**	**[-0.2062, 0.0212]**

3.1. Inter-Run Reproducibility: Mean Cq Values Experiment 1 vs Experiment 2

**Species**	**Genotype**	**Treatment**	**Exp 1 Mean Cq**	**Exp 2 Mean Cq**	**Δ Cq |E1-E2|**
A. thaliana	**Control**	WT	19.837	19.440	0.397
		SO OE	20.190	19.763	0.427
		SO Ri	20.267	20.495	0.228
		apr2 KO	20.320	20.195	0.125
		apr2 KO/*SO* OE	19.917	19.483	0.434
		SlSiR OE/*SO* OE	19.973	19.993	0.020
		SiR OE^{C2}+SO OE^{C2}_1	20.133	19.755	0.378
		SiR OE^{C2}+*SO* OE^{C2}_2	19.530	19.570	0.040
		SiR OE^{C4}/*SO* OE_1	19.797	19.733	0.064
		SiR OE^{C4}/*SO* OE_2	19.877	19.960	0.083
	**Drought**	WT	20.180	19.770	0.410
		SO OE	20.063	20.048	0.015
		SO Ri	19.655	19.438	0.217
		apr2 KO	20.038	20.170	0.132
		apr2 KO/*SO* OE	20.140	20.128	0.012
		SlSiR OE/*SO* OE	20.048	20.175	0.127
		SiR OE^{C2}+SO OE^{C2}_1	20.280	20.303	0.023
		SiR OE^{C2}+*SO* OE^{C2}_2	20.190	20.230	0.040
		SiR OE^{C4}/*SO* OE_1	20.180	20.150	0.030
		SiR OE^{C4}/*SO* OE_2	20.473	20.508	0.035
**Tomato**	**Control**	RR (WT)	20.592	20.477	0.115

		SO OE	19.997	20.407	0.410
		SiR OE	20.252	20.288	0.036
		SiR OE/*SO* OE	20.683	20.363	0.320
	**Drought**	RR (WT)	21.231	21.470	0.239
		SO OE	21.340	21.410	0.070
		SiR OE	21.764	21.688	0.076
		SiR OE/*SO* OE	22.060	21.723	0.337

3.2. TOST equivalence test - Inter-Run Reproducibility (Experiment 1 vs Experiment 2)

	**Parameter**	**A. thaliana Control**	**A. thaliana Drought**	**Tomato Control**	**Tomato Drought**
**Sample**	n per group (Control / Drought)	10	10	4	4
**Descriptive Statistics**	**Mean Difference E1-E2 (Δ Cq)**	**0.1454**	**0.0327**	**-0.0028**	**0.0260**
	SD of Differences	0.2450	0.1642	0.3082	0.2440
	Max |Δ Cq|	0.434	0.410	0.410	0.337
	Standard Error (SE)	0.0775	0.0519	0.1541	0.1220
	Degrees of Freedom (df)	9	9	3	3
**Equivalence Parameters**	Equivalence Bound (+/-Δ)	+/-0.5 Cq	+/-0.5 Cq	+/-0.5 Cq	+/-0.5 Cq
	Fold-change Threshold	1.414	1.414	1.414	1.414
**TOST Statistics**	Test 1 t-statistic (diff > -0.5)	8.3293	10.2587	3.2266	4.3108
	**Test 1 p-value**	**8.01×10^{-6}**	**1.45×10^{-6}**	**2.42×10^{-2}**	**1.15×10^{-2}**
	Test 2 t-statistic (diff < +0.5)	4.5763	8.9993	3.2623	3.8847
	**Test 2 p-value**	**6.67×10^{-4}**	**4.27×10^{-6}**	**2.35×10^{-2}**	**1.51×10^{-2}**
**Confidence Interval**	t-critical (95th percentile)	1.8331	1.8331	2.3534	2.3534
	**90% CI of Mean Difference**	**[0.0034, 0.2874]**	**[-0.0625, 0.1279]**	**[-0.3654, 0.3599]**	**[-0.2612, 0.3132]**

Supplementary Protocol: Generation of overexpressed SiR and SO in *Arabidopsis* by CRISPR technology

Arabidopsis plants over expressed *SiR* and *SO* were generated using multiplexed CRISPR-Act 3.0 Systems (Pan et al., 2021). Construct 1 has pYPQ144+ SiRG1 SiRG2 SOG1 SOG2 as gRNA entry vector, dzCAS9 Act3 as Cas entry vector and pYPQ202 as destination vector. Construct 2 has pYPQ144+ SiRG1 SiRG1 SiRG3 SiRG4 as gRNA entry vector, dzCAS9 Act3 as Cas entry vector and pYPQ202 as destination vector. For the generation of double mutant two construct has been outsourced from GenScript, USA.

Construction of vector construct 1 (pYPQ202-pdzCas9-Act3-SiR-gR1,gR2, SO- gR1,gR2): First, oligos corresponding to *SiR*-gR1 (*SiR*-gR1-forward and *SiR*-gR1-reverse), *SiR*-gR2 (*SiR*-gR2-forward and *SiR*-gR2-reverse), *SO*-gR1 (*SO*-gR1-forward and *SO*-gR1-reverse) and *SO*-gR2 (*SO*-gR2-forward and *SO*-gR2-reverse) phosphorylated and annealed, and cloned into Esp3I-linearized pYPQ131B2.0, pYPQ132B2.0, pYPQ133B2.0 and pYPQ134B2.0 respectively (step 1). These four vectors mixed with pYPQ144 for a golden gate reaction to make pYPQ144-*SiR*-gRNA-1,2-SO-gRNA1,2 (step 2). Finally, this gRNA entry clone was mixed with dzCas9-ACT3 and pYPQ202 in a multisite Gateway reaction to make pYPQ202-dZCas9- ACT3- *SIR*-gRNA-1,2-SO-gRNA1,2 (step 3).

Construction of Vector construct 2 pYPQ202-pdzCas9-Act3-SiR-gR1,gR2, gR3,gR4): First, oligos corresponding to *SiR*-gR1 (*SiR*-gR1-forward and *SiR*-gR1-reverse), *SiR*-gR2 (*SiR*-gR2-forward and *SiR*-gR2-reverse), *SiR*-gR3 (*SiR*-gR3-forward and *SiR*-gR3-reverse) and *SiR*-gR2 (*SiR*-gR4-forward and *SiR*-gR4-reverse), phosphorylated and annealed, and cloned into Esp3I-linearized pYPQ131B2.0, pYPQ132B2.0, pYPQ133B2.0 and pYPQ134B2.0, respectively (step 1). These four vectors mixed with pYPQ144 for a golden gate reaction to make pYPQ144-*SiR*-gRNA-1,2,3,4 (step 2). Finally, this gRNA entry clone was mixed with dzCas9-ACT3 and pYPQ202 in a multisite gateway reaction to make pYPQ202-dZCas9- ACT3- *SiR*-gRNA-1,2,3,4 (step 3). Primer sequences used for both construct preparation is shown in **Table S2**. The constructs were introduced into *Agrobacterium tumefaciens* strain GV3101 by electroporation and transformed into *Arabidopsis* ecotype Col-0 plants using the floral dip method (Clough and Bent, 1998). Transformed lines were selected by resistance to hygromycin (sigma) for CRISPR plants and confirmed by western blotting for homozygous lines.
